# Mindfulness-based programmes for mental health promotion in adults in non-clinical settings: protocol of an individual participant data meta-analysis of randomised controlled trials

**DOI:** 10.1136/bmjopen-2021-058976

**Published:** 2022-04-10

**Authors:** Julieta Galante, Claire Friedrich, Tim Dalgleish, Ian R White, Peter B Jones

**Affiliations:** 1 Department of Psychiatry, University of Cambridge, Cambridge, UK; 2 NIHR Applied Research Collaboration East of England, Cambridge, UK; 3 MRC Cognition and Brain Sciences Unit, University of Cambridge, Cambridge, UK; 4 Cambridgeshire and Peterborough NHS Foundation Trust, Cambridge, UK; 5 MRC Clinical Trials Unit, University College London, London, UK

**Keywords:** mental health, complementary medicine, public health

## Abstract

**Introduction:**

With mental ill health listed as a top cause of global disease burden, there is an urgent need to prioritise mental health promotion programmes. Mindfulness-based programmes (MBPs) are being widely implemented to reduce stress in non-clinical settings. In a recent aggregate-level meta-analysis we found that, compared with no intervention, these MBPs reduce average psychological distress. However, heterogeneity between studies impedes generalisation of effects across every setting. Study-level effect modifiers were insufficient to reduce heterogeneity; studying individual-level effect modifiers is warranted. This requires individual participant data (IPD) and larger samples than those found in existing individual trials.

**Methods and analysis:**

We propose an IPD meta-analysis. Our primary aim is to see if, and how, baseline psychological distress, gender, age, education and dispositional mindfulness moderate the effect of MBPs on distress. We will search 13 databases for good-quality randomised controlled trials comparing in-person, expert-defined MBPs in non-clinical settings with passive controls. Two researchers will independently select, extract and appraise trials using the revised Cochrane risk-of-bias tool. Anonymised IPD of eligible trials will be sought from authors, who will be invited to collaborate.

The primary outcome will be psychological distress measured using psychometrically validated questionnaires at 1–6 months after programme completion. Pairwise random-effects two-stage IPD meta-analyses will be conducted. Moderator analyses will follow a ‘deft’ approach. We will estimate subgroup-specific intervention effects. Secondary outcomes and sensitivity analyses are prespecified. Multiple imputation strategies will be applied to missing data.

**Ethics and dissemination:**

The findings will refine our knowledge on the effectiveness of MBPs and help improve the targeting of MBPs in non-clinical settings. They will be shared in accessible formats with a range of stakeholders. Public and professional stakeholders are being involved in the planning, conduct and dissemination of this project.

**PROSPERO registration number:**

CRD42020200117.

**Strengths and limitations of this study**

- This is, to our knowledge, the first individual participant data (IPD) meta-analysis assessing the effectiveness of mindfulness-based programmes to reduce psychological distress among adults in non-clinical settings, and how it varies as a function of individual differences.
- Preceded by a comprehensive systematic review, this IPD meta-analysis will have greater statistical power to detect effect modifiers than any of the individual trials.
- This IPD meta-analysis can overcome some, but not all, of the existing trials’ methodological shortcomings.
- As a secondary-data analysis, this study depends on trial data being shared; this factor can limit the validity and generalisability of the findings.
- The outcomes and effect modifiers that can be assessed are limited to those that the existing trials have measured, and how they have measured them.

## Introduction

Common mental health disorders such as depression are among the top worldwide causes of morbidity, generating a very significant burden on societies.^1^ The COVID-19 pandemic, a global natural stressor, is increasing this burden.^2^ The last decade has seen an expansion of mental health prevention and promotion programmes in workplaces, educational establishments and other community settings.^3^ They usually target psychological distress, a concept encompassing a range of disturbing or unpleasant mental or emotional experiences which, if unaddressed, can result in mental and physical health disorders.^4^

Frequently promoted as a universal tool to reduce stress,^5^ mindfulness-based programmes (MBPs) are among the most commonly implemented preventive activities.^6^ In the USA, mindfulness training is present at 79% of medical schools,^7^ and offered by 22% of employers.^8^ MBPs typically define mindfulness as ‘the awareness that emerges through paying attention on purpose, in the present moment and non-judgmentally to the unfolding of experience moment by moment’.^9^ Their core elements are an emphasis on teaching mindfulness meditation and mindful activities, scientific approaches to managing health, suitability for delivery in public institutions across a range of settings and cultures and class-based experiences of collective and individual inquiry with a qualified teacher in a participatory learning process.^10^

We recently completed a systematic review and aggregate-level meta-analysis of randomised controlled trials (RCTs) assessing MBPs for mental health promotion in adults in non-clinical settings (from now on referred to as our previous review).^11^ We found that, compared with no intervention, MBPs of the included studies, on average, reduced psychological distress (our most measured and robust outcome). However, given the heterogeneity between studies, the findings did not support generalisation of MBP effects across every setting. We investigated study-level factors that could moderate the effect of the MBPs on psychological distress, such as programme characteristics or type of population being targeted, but these were not able to fully explain the seen heterogeneity. Participant-level effect modifiers, such as participants’ prior mental health, may be at play.

Individual participant data (IPD) meta-analyses are the only practical choice for exploring how MBP effectiveness varies as a function of individual differences.^12–14^ In IPD meta-analyses, rather than extracting summary data from trial publications, the original individual-level trial data are sought directly from trial authors. Aggregate-level meta-analyses (the most common ones) may give misleading individual-level moderator results because of study-level confounding.^14^ Conducting further RCTs to perform subgroup analyses is expensive and impractical due to the large sample sizes required, particularly to find small-to-moderate effect sizes in low-risk populations. It is notoriously difficult to identify genuine predictors of differential response from single trials, as there is high potential for type I and type II errors.^14^ IPD meta-analyses can obtain results for specific subgroups of participants across studies, and differential effects can be assessed across individuals, which can help reduce between-study heterogeneity.^15^ Other advantages of this approach are that data can be checked and re-analysed, and missing data can be accounted for at the individual level.^16^ Finally, they can act as a stimulus for international collaboration, debate and consensus, and form the basis for further data sharing and open research. A key limitation of IPD meta-analyses is that the outcomes and effect modifiers that can be assessed are limited to those that the existing trials have measured, and how they have measured them. IPD meta-analyses also depend on trial authors’ willingness to share data, and on how well the trials were conducted.

### The role of individual differences in MBPs

Preliminary evidence strongly suggests that the effectiveness of MBPs vary as a function of individual differences.^17^ There have been several calls to study MBP effect modification more extensively, and small sample sizes have frequently been cited as a limiting factor.^12 13 18–21^

Individuals with worse mental health to begin with may be the most likely to benefit because there is more room to what can be learnt. There is evidence that MBPs targeted at stressed groups,^11 22^ those with anxiety or mood disorders,^23^ those with higher symptom severity^24 25^ or those experiencing stressful times^26^ have larger effects. An IPD meta-analysis of mindfulness-based cognitive therapy (MBCT) to prevent recurrent depression relapse found a significant relative reduction in effect with better baseline status.^27^ Most findings thus suggest that higher baseline distress levels strengthen intervention effects, although some have found no evidence of interaction.^28^

A meta-analysis of workplace MBPs found a significant moderating effect of gender on well-being and life satisfaction.^29^ This finding adds to previous evidence suggesting that MBP effects on men are smaller than those on women.^20 30^ It has been posited that women tend to internalise their distress more, which may make techniques such as mindfulness work more favourably, while an externalising coping style, more frequently associated with men, may limit the effectiveness of MBPs.^20^ Others proposed that neuroticism and conscientiousness, personality factors more common among women, may amplify MBP effects.^20 30 31^ Some studies exploring gender as an effect modifier, including the MBCT IPD meta-analysis, have found no moderating effects.^22 27^

Meta-analytic evidence suggests that MBPs for children and adolescents^32^ and university students,^33^ have larger effects than those for adults.^11^ While some studies reported no moderating effects of age, one study found age to moderate intervention effects on levels of anxiety, with older adults reporting smaller reductions in anxiety over time compared with their younger counterparts.^34^ At play may be cognitive and cultural factors that are intrinsic to age such as plasticity and curiosity, or confounders such as education (eg, young people belong to university student samples). However, age was not an effect modifier in the MBCT IPD meta-analysis and other studies.^27 35^

Education levels are known to moderate the effectiveness of some psychological interventions.^36^ Concerns have been voiced that current MBPs may not be inclusive of diverse education backgrounds because of their language and cultural references.^37^ A recent meta-analysis has reported significant moderating effects of level of education in workplace MBPs, finding a larger improvement in well-being among more highly educated participants.^29^ However, education was not an effect modifier in the MBCT IPD meta-analysis.^27^

Baseline levels of dispositional mindfulness, a multidimensional construct reﬂecting an individual’s focus and quality of their attention,^38^ may moderate MBP effects, but the evidence is inconsistent and shows a complex picture.^39^ For example, Shapiro *et al* reported that participants with higher trait mindfulness at baseline experienced greater and long-lasting improvements in well-being and distress,^21^ while Greeson *et al* found that baseline dispositional mindfulness did not moderate the effect of an MBP on depressive symptoms.^35^ A higher level of dispositional mindfulness may be needed to engage with MBPs, but this may also limit the amount that is to be learnt.

With the proliferation of mindfulness provision in recent times, understanding what works, for whom and in what circumstances becomes a pressing issue. This information is essential to tailor interventions, maximising effectiveness, cost-effectiveness and ensuring intervention harm minimisation.^40^

We plan to conduct a systematic review and individual participant data meta-analysis to answer the following main research question: Do selected participant-level characteristics moderate the effect of mindfulness-based programmes (MBPs) on psychological distress among adults in non-clinical settings, and if so, how do they do it? Our main aim is to see whether and how baseline psychological distress, gender, age, education and dispositional mindfulness moderate the effect of MBPs on psychological distress compared with no intervention. Effect modifiers for this IPD meta-analysis have been selected based on existing theories and empirical evidence, and on availability as they are commonly reported among trials and are comparable across international samples. Exploring these potential effect modifiers with IPD will address current limitations and support our understanding of individual differences in response to MBPs.^41^

## Methods and analysis

This protocol follows Preferred Reporting Items for Systematic Reviews and Meta-Analyses Protocols guidelines.^42^

### Study search and selection

The search will update that of our previous review.^11^ Thirteen databases will be included: Allied and Complementary Medicine, Applied Social Sciences Index and Abstracts, the Cochrane Central Register of Controlled Trials, the Cumulative Index to Nursing and Allied Health Literature, Education Resources Information Center, Electronic Theses Online Service, Excerpta Medica Database (EMBASE), Medical Literature Analysis and Retrieval System Online, ProQuest, PsycINFO, Scopus, Web of Science and WHO International Clinical Trials Registry Platform. Predefined search strategies using keywords and controlled vocabulary will be adapted and applied to each database. Where possible the search terms mindful and meditation will be combined with a pretested, sensitive filter for RCTs,^43^ otherwise they will be combined with “randomize”, “RCT”, “random allocation” and “random assignment”. Search terms will be modified to include truncation, proximity indicators and wild cards. Additionally, when applicable, subject headings will be exploded. The database search strategy for EMBASE is available in online supplemental appendix 1 as an example; all the strategies are also available in the publication of our previous review.^11^ In addition to the electronic search, we will inspect the reference lists of identified RCTs and reviews. No language limitations will be included. Non-public sources of studies will not be used in the searches,^44^ but authors will be contacted to provide information as outlined herein.

10.1136/bmjopen-2021-058976.supp1 Supplementary data

The review inclusion criteria are presented in table 1. These are similar but narrower in scope than our previous review in order to produce a more focused and better-quality analysis, and because it is infeasible for us to collect IPD from the 136 RCTs included in that review. Online MBPs were excluded because we believe they are different enough from in-person MBPs (e.g., typically not group-based, and fully or semi-automated) to merit their own separate analysis.^45^

**Table 1:** Review inclusion criteria

Study aspect	Inclusion criterion
Design	Parallel-arm RCTs including cluster RCTs.
Intervention	Group-based first-generation MBPs as defined by Crane *et al*,^10^ with a minimum intensity of four 1-hour in-person teacher-led sessions or equivalent*.
Comparison	Passive control groups such as no intervention, waitlists or treatment-as-usual if the MBP arm also had access to it.
Population	Adult (aged 18+ years) participants living in the community, as long as the trial had not selected them for having any particular clinical condition. MBPs targeting specific community groups were included. Trials with slightly younger participants (e.g., those in university settings where some students will turn 18 during the first academic year) will be included.
Outcomes	Self-reported psychological distress measured between 1 and 6 months after MBP completion.
Effect modifiers	At least one of the following has been measured: baseline psychological distress, gender, age, education and dispositional mindfulness.
Quality	A maximum of two high risk-of-bias sources as assessed using the RoB2 tool^47^ before obtaining IPD (rationale in ‘Risk-of-bias assessment’ section).

*Four MBP sessions were used as the ‘minimum dose’ for participants in previous studies,^59^ and 1 hour sessions are common in non-clinical settings.^60^

IPD, individual participant data; MBP, mindfulness-based programme; RCT, randomised controlled trial; RoB2, risk-of-bias tool.

Trials included in our previous review and studies found through the search update will be assessed for inclusion in this IPD meta-analysis. Two researchers will independently review the titles and abstracts of all records retrieved by the search. If both reviewers agree that a record does not meet eligibility criteria, it will be excluded. The full text of all remaining records will be obtained, and the same eligibility criteria will be applied to them by the two reviewers for a final selection. Disagreements will be decided via consensus between two senior team members (TD and PBJ) blind to trial results.

#### Data collection and processing

Two reviewers will independently extract study-level characteristics of newly identified studies into extraction forms similar to those used in our previous review (online supplemental appendix 2). Authors of eligible studies will be invited to collaborate. Publication co-authorship, help with data preparation and transfer, and secure and confidential data management will be offered. If necessary, authors other than the correspondent author will be contacted. IPD will be considered unavailable where no authors have responded to multiple contact attempts, where authors indicate that they no longer have access to the data, or where authors decline to participate.

Anonymised trial IPD relevant to the analyses proposed herein will be requested from authors who accept our invitation. We will request IPD for all randomised participants, independently of whether trial publications used all of the data or only a fraction. We will prefer datasets without imputed missing data.

Participant-level data characteristics will be checked as follows using structured forms. IPD from each trial will be checked for missing participants (eg, compare IPD samples against trial Consolidated Standards of Reporting Trials diagrams to ensure that IPD from all randomised participants is included if available), for missing outcomes and missing prespecified effect modifiers (compared against trial publications and protocols), and for invalid, out of range or inconsistent items (eg, unusually old or young participants), before being converted to standard format. We will request individual items from questionnaires, recalculating scale-specific scores where possible. We will check with trial authors whether any questionnaire items had been reversed, if applicable. IPD will be cross-examined against the summary statistics reported in trial publications. Inconsistencies will be checked by another reviewer. If they confirm that the numbers do not match, we will attempt to explain the difference (eg, the publication may have used a per-protocol sample and we may have used the full randomised sample), and we will contact trial authors for clarification until inconsistencies are understood, and corrected if applicable.

#### Risk-of-bias assessment

Two reviewers will independently assess newly found trials’ risk of bias using the revised Cochrane risk-of-bias tool (RoB2) for randomised trials applied to the outcomes included in this review.^46 47^ This tool stringently measures potential bias across five sources: (1) randomisation, (2) deviations from intended interventions, (3) missing outcome data, (4) measurement of the outcome and (5) selection of the reported result. We will resolve discrepancies through discussion, involving a third reviewer where necessary.

Our previous review has found that many trials have high risk of bias from several sources, reducing confidence in the cumulative evidence. To understand how results were affected, we performed a sensitivity analysis removing trials deemed to be at high risk of bias from three or more sources, which divided the sample into roughly equal parts. The sensitivity analysis showed that the results were generally sensitive to this criterion. Accordingly, to maximise confidence in the IPD meta-analysis results and to maintain consistency with our previous review, we plan to only include trials with a maximum of two high risk-of-bias domains, as assessed before obtaining IPD. These are the trials likely to provide the most reliable evidence in the field.

We acknowledge, however, that this criterion is suboptimal, potentially imposing a limitation on our findings. The RoB2 tool has not been validated as a scale, so there are no validated cut-off points and domains may not be interchangeable.^48^ Therefore, the included studies may have very different types of flaws. These flaws will be described through a detailed assessment of the risks of bias of each of the included studies using the RoB2 tool. We will also discuss our findings in relation to the sensitivity analysis performed in our previous review.

Once studies have been selected and IPD obtained, risk of bias for individual studies will be updated according to the IPD available (eg, risk lowered if IPD includes participants missing in published trial reports). We will check allocation for any unusual patterns. When key aspects are unclear, we will seek information from study authors. In order to assess the confidence in the cumulative evidence, we will use the Grading of Recommendations, Assessment, Development and Evaluations approach Guyatt *et al* 2008.^49^

### Effect measures

The main outcome will be self-reported psychological distress measured between 1 and 6 months after programme completion using psychometrically valid questionnaires scored on a continuous scale (eg, Perceived Stress Scale, General Health Questionnaire, Depression, Anxiety and Stress Scale). Questionnaires asking about fleeting states (eg, “How do you feel now?”) will be excluded.

Postintervention psychological distress measures (ie, those taken <1 month after programme completion) will be grouped and considered as a secondary outcome: they do not inform stable changes, therefore are less useful for understanding the real-life impact of MBPs. Psychological distress follow-up measured beyond 6 months will be grouped and also considered as a secondary outcome. If a study measured the outcome more than once within the time point range of interest, the longest follow-up will be used.

We expect that trials will use different questionnaires to measure psychological distress, therefore we will standardise them using z-scores. We will calculate the the analysis of covariance estimate (final score adjusted for baseline score) and adjust it for the available prespecified effect modifiers.^50 51^

If a trial reports more than one psychological distress measure within the same time point range, we will prefer the one assigned as primary outcome by the trialists; if this is not stated or none are primary outcomes, the one with best psychometric properties; if they have similar properties, the one that is used most frequently in the other studies. Full questionnaire scales and untransformed data will be preferred.

### Data synthesis

Although this project focuses on the effect moderator analyses, overall effects will be calculated and will be compared with those found in our previous review. Data synthesis will be quantitative. Two-stage IPD meta-analyses will be performed, as they automatically stratify parameter estimates by trial, use well‐known meta‐analysis methods, are more transparent than one-stage methods, and easily enable forest plots.^50^ They will be univariate for the time-point ranges for which data from all the trials are available, otherwise they will be multivariate and include all available time-point ranges.^52^

Stage one of the two-stage IPD meta-analyses will involve conducting linear regressions separately by trial to estimate the trial’s intervention effects. The models will include the baseline measurement of the outcome and the prespecified effect modifiers available for that trial.^50^ Stage 2 will combine the intervention effects from each trial using pairwise random-effects meta-analyses (a common effect is highly implausible) within comparator categories.

The main analysis will compare MBPs with a combination of all the passive control groups. If the included trials also compared MBPs with other interventions, these will be grouped under the comparator ‘active control’, and effects will be explored for this comparison in secondary analyses. In the event of finding multi-armed trials with multiple control groups that fit one category, these control groups will be combined. Two-arm trials that compare two eligible MBPs with each other will not be included. In multi-arm trials that do this, the two MBP arms will be combined for meta-analysis.

Estimation of heterogeneity will be performed using restricted maximum likelihood. To quantify the heterogeneity in the intervention effect, approximate prediction intervals will be calculated.^53^ Intention-to-treat analyses of individual trials will be conducted for verification, to compare against published analyses and to discuss reasons for potential differences. Trials for which IPD are not made available will be included in a sensitivity analysis incorporating the available aggregate data. Results will be compared with IPD-data-only results.^16^

Multiple imputation strategies will be applied to missing data (details in online supplemental appendix 3).^50 54 55^ A sensitivity analysis will compare results of imputed datasets with observed datasets. We will assess departures of the data missing at random assumption in sensitivity analyses at the individual study level, modelling missing data as 10% and 20% worse psychological stress scores than observed data. We will also explore the scenarios of missing distress scores in the intervention arm being worse than passive control group scores. In the mindfulness group, participants who felt worse may have been less willing to answer because they were expecting an improvement or thought that they had done something wrong. Instead, passive control group participants may have expected to feel worse. We will explore how much worse missing outcome scores in the mindfulness arms would need to be for the significance and direction of the intervention effect to change.

### Moderator analyses

The main moderator analyses will look at the effect of the moderators of interest one by one; if multiple interaction effects are found we will explore multivariable options to adjust for confounding as a secondary analysis. For each of the main moderator analyses, a treatment by participant covariate interaction term will be incorporated in the intervention effect trial regression models (first stage of two-stage meta-analysis), and the estimated interactions will be combined in a random effects meta-analysis. This method, known as the ‘deft’ approach, will account for clustering of participants and separate out within-study and across-study information, avoiding ecological bias.^56 57^

We will estimate subgroup-specific intervention effects by repeating the analysis procedure with the interaction parameters fixed at their ‘deft’ estimates. Trials and/or individuals with missing values on an effect modifier will be excluded from the estimation of that interaction. If we find interaction effects after confounding adjustment, we will present a predictive model. We will test whether there is evidence of non-linear effects; if we find such evidence we will explore non-linear models.^56 58^

Continuous variables will not be categorised for analysis. We expect that trials will use different questionnaires to measure baseline psychological distress and dispositional mindfulness; we will standardise them using z-scores. Education level data are usually collected in the form of categories with a natural ordering; if that is the case then a linear trend across categories will be assumed.^57^ Where trials have used different categories for collecting education level data, we shall strive to retain an ordered-categorical approach where levels have been collapsed by, for instance, PhD=1, BA=2, PhD/BA=1.5 or by estimating years of education. Genders other than man/woman will be combined into an ‘other’ category.

### Ethics and dissemination

No local ethics approval was deemed necessary for this project following consultation with the research governance team. Trial authors will be requested to anonymise datasets prior to sharing them, and asked to confirm they have obtained ethical approval for sharing trial data anonymously. Data management and analysis will take place at the Department of Psychiatry, University of Cambridge. Data as obtained from individual trial authors will be stored at the highly secure Clinical School Secure Data Hosting Service and checked for any residual identifiable data before making copies to be used in normal workstations. The aggregate data and analysis code will be shared in a public repository.

Findings will be disseminated within the academic community through publication in scientific journals, conference presentations and networking. Professional stakeholders will be reached through activities focused on discussing the applicability of the findings. Media channels, social media (@MSSatUoC) and a variety of presentation formats will be used to engage with the wider public.

### Patient and public involvement

A public stakeholder group is providing input throughout the life of this project. Members bring experiential expertise on mindfulness’ effects and how they interact with contextual or personal factors, and on mental health promotion in daily life. We train and support them so that they are able to conceptually understand the study and can co-produce it. Stakeholders shaped the research questions and prioritised outcomes and moderation analyses. They will be invited to contribute to the day-to-day research process as research partners, for example, by selecting studies and extracting data. They will be involved in interpreting the results, creating an impact plan and disseminating the findings. We are also involving a group of professional stakeholders.

## Supplementary Material

Reviewer comments

Author's manuscript

## Data Availability

Data sharing not applicable as no datasets generated and/or analysed for this study.

## References

[1] Global Burden of Disease Study 2013 Collaborators. Global, regional, and national incidence, prevalence, and years lived with disability for 301 acute and chronic diseases and injuries in 188 countries, 1990-2013: a systematic analysis for the global burden of disease study 2013. Lancet 2015;386:743–800. 10.1016/S0140-6736(15)60692-4 26063472 PMC4561509

[2] Vindegaard N, Benros ME. COVID-19 pandemic and mental health consequences: systematic review of the current evidence. Brain Behav Immun 2020;89:531–42. 10.1016/j.bbi.2020.05.048 32485289 PMC7260522

[3] Samele C. Increasing momentum in prevention of mental illness and mental health promotion across Europe. BJPsych Int 2016;13:22–3. 10.1192/s2056474000000957 29093889 PMC5618895

[4] Russ TC, Stamatakis E, Hamer M, et al. Association between psychological distress and mortality: individual participant pooled analysis of 10 prospective cohort studies. BMJ 2012;345:e4933. 10.1136/bmj.e4933 22849956 PMC3409083

[5] Kabat-Zinn J. Foreword: seeds of a necessary global renaissance in the making: the refining of psychology's understanding of the nature of mind, self, and embodiment through the lens of mindfulness and its origins at a key inflection point for the species. Curr Opin Psychol 2019;28:xi-xvii. 10.1016/j.copsyc.2019.02.005 31097398

[6] Burke A, Lam CN, Stussman B, et al. Prevalence and patterns of use of MANTRA, mindfulness and spiritual meditation among adults in the United States. BMC Complement Altern Med 2017;17:316. 10.1186/s12906-017-1827-8 28619092 PMC5472955

[7] Barnes N, Hattan P, Black DS, et al. An examination of Mindfulness-Based programs in US medical schools. Mindfulness 2017;8:489–94. 10.1007/s12671-016-0623-8

[8] Fortune. Meditation has become a Billion-Dollar business, 2016. Available: http://fortune.com/2016/03/12/meditation-mindfulness-apps/ [Accessed 01 Dec 2016].

[9] Kabat-Zinn J. Full catastrophe living, revised edition: how to cope with stress, pain and illness using mindfulness meditation. 2 edn. London: Piatkus, 2013.

[10] Crane RS, Brewer J, Feldman C, et al. What defines mindfulness-based programs? the warp and the weft. Psychol Med 2017;47:990–9. 10.1017/S0033291716003317 28031068

[11] Galante J, Friedrich C, Dawson AF, et al. Mindfulness-based programmes for mental health promotion in adults in nonclinical settings: a systematic review and meta-analysis of randomised controlled trials. PLoS Med 2021;18:e1003481. 10.1371/journal.pmed.1003481 33428616 PMC7799763

[12] Goldberg SB, Sun S, Davidson RJ. The empirical status of mindfulness based interventions: a systematic review of 44 meta-analyses of randomized controlled trials. Perspect Psychol Sci 2020;17. 10.1177/1745691620968771 PMC8364929 33593124

[13] Vibe M, Bjørndal A, Tipton E, et al. Mindfulness based stress reduction (MBSR) for improving health, quality of life, and social functioning in adults. Campbell Syst Rev 2012;8:1–127. 10.4073/csr.2012.3

[14] Hingorani AD, Windt DAvander, Riley RD, et al. Prognosis research strategy (progress) 4: stratified medicine research. BMJ 2013;346:e5793. 10.1136/bmj.e5793 23386361 PMC3565686

[15] Ioannidis JP, Lau J, Pearls U. Uncontrolled pearls, controlled evidence, meta-analysis and the individual patient. J Clin Epidemiol 1998;51:709–11. 10.1016/S0895-4356(98)00042-0 9743320

[16] Riley RD, Lambert PC, Abo-Zaid G. Meta-Analysis of individual participant data: rationale, conduct, and reporting. BMJ 2010;340:c221. 10.1136/bmj.c221 20139215

[17] Keng S-L, Smoski MJ, Robins CJ. Effects of mindfulness on psychological health: a review of empirical studies. Clin Psychol Rev 2011;31:1041–56. 10.1016/j.cpr.2011.04.006 21802619 PMC3679190

[18] Davidson RJ. Mindfulness-Based cognitive therapy and the prevention of depressive relapse: measures, mechanisms, and mediators. JAMA Psychiatry 2016;73:547–8. 10.1001/jamapsychiatry.2016.0135 27119526

[19] Ospina MB, Bond K, Karkhaneh M, et al. Meditation practices for health: state of the research. Evid Rep Technol Assess 2007:1–263. PMC4780968 17764203

[20] Rojiani R, Santoyo JF, Rahrig H, et al. Women benefit more than men in response to College-based meditation training. Front Psychol 2017;8:551. 10.3389/fpsyg.2017.00551 28473783 PMC5397480

[21] Shapiro SL, Brown KW, Thoresen C, et al. The moderation of Mindfulness-based stress reduction effects by trait mindfulness: results from a randomized controlled trial. J Clin Psychol 2011;67:267–77. 10.1002/jclp.20761 21254055

[22] Schellekens MPJ, van den Hurk DGM, Prins JB, et al. Mindfulness-based stress reduction added to care as usual for lung cancer patients and/or their partners: a multicentre randomized controlled trial. Psychooncology 2017;26:2118–26. 10.1002/pon.4430 28337821

[23] Hofmann SG, Sawyer AT, Witt AA, et al. The effect of mindfulness-based therapy on anxiety and depression: a meta-analytic review. J Consult Clin Psychol 2010;78:169–83. 10.1037/a0018555 20350028 PMC2848393

[24] Roos CR, Bowen S, Witkiewitz K. Baseline patterns of substance use disorder severity and depression and anxiety symptoms moderate the efficacy of mindfulness-based relapse prevention. J Consult Clin Psychol 2017;85:1041–51. 10.1037/ccp0000249 29083220 PMC5679292

[25] Khoury B, Lecomte T, Fortin G, et al. Mindfulness-based therapy: a comprehensive meta-analysis. Clin Psychol Rev 2013;33:763–71. 10.1016/j.cpr.2013.05.005 23796855

[26] Galante J, Stochl J, Dufour G, et al. Effectiveness of providing university students with a mindfulness-based intervention to increase resilience to stress: 1-year follow-up of a pragmatic randomised controlled trial. J Epidemiol Community Health 2021;75:151–60. 10.1136/jech-2020-214390 32913130 PMC7116569

[27] Kuyken W, Warren FC, Taylor RS, et al. Efficacy of Mindfulness-Based cognitive therapy in prevention of depressive relapse: an individual patient data meta-analysis from randomized trials. JAMA Psychiatry 2016;73:565–74. 10.1001/jamapsychiatry.2016.0076 27119968 PMC6640038

[28] Fiocco AJ, Mallya S, Farzaneh M, et al. Exploring the benefits of mindfulness training in healthy community-dwelling older adults: a randomized controlled study using a mixed methods approach. Mindfulness 2019;10:737–48. 10.1007/s12671-018-1041-x

[29] Vonderlin R, Biermann M, Bohus M, et al. Mindfulness-Based programs in the workplace: a meta-analysis of randomized controlled trials. Mindfulness 2020;11:1579–98. 10.1007/s12671-020-01328-3

[30] de Vibe M, Solhaug I, Tyssen R, et al. Mindfulness training for stress management: a randomised controlled study of medical and psychology students. BMC Med Educ 2013;13:107. 10.1186/1472-6920-13-107 23941053 PMC3751423

[31] Galante J, Dufour G, Vainre M, et al. A mindfulness-based intervention to increase resilience to stress in university students (the mindful student study): a pragmatic randomised controlled trial. Lancet Public Health 2018;3:e72–81. 10.1016/S2468-2667(17)30231-1 29422189 PMC5813792

[32] Dunning DL, Griffiths K, Kuyken W, et al. Research Review: The effects of mindfulness-based interventions on cognition and mental health in children and adolescents - a meta-analysis of randomized controlled trials. J Child Psychol Psychiatry 2019;60:244–58. 10.1111/jcpp.12980 30345511 PMC6546608

[33] Dawson AF, Brown WW, Anderson J, et al. Mindfulness-Based interventions for university students: a systematic review and meta-analysis of randomised controlled trials. Appl Psychol Health Well Being 2020;12:384–410. 10.1111/aphw.12188 31743957

[34] Nyklíček I, Irrmischer M. For whom does Mindfulness-Based stress reduction work? moderating effects of personality. Mindfulness 2017;8:1106–16. 10.1007/s12671-017-0687-0 28757903 PMC5506177

[35] Greeson JM, Smoski MJ, Suarez EC, et al. Decreased symptoms of depression after mindfulness-based stress reduction: potential moderating effects of religiosity, spirituality, trait mindfulness, sex, and age. J Altern Complement Med 2015;21:166–74. 10.1089/acm.2014.0285 25695903 PMC4365440

[36] Ebert DD, Donkin L, Andersson G, et al. Does Internet-based guided-self-help for depression cause harm? an individual participant data meta-analysis on deterioration rates and its moderators in randomized controlled trials. Psychol Med 2016;46:2679–93. 10.1017/S0033291716001562 27649340 PMC5560500

[37] Russell L, Ugalde A, White V, et al. Relevance of mindfulness practices for culturally and linguistically diverse cancer populations. Psychooncology 2019;28:2250–2. 10.1002/pon.5221 31487412

[38] Rau HK, Williams PG. Dispositional mindfulness: a critical review of construct validation research. Pers Individ Dif 2016;93:32–43. 10.1016/j.paid.2015.09.035

[39] Gawrysiak MJ, Grassetti SN, Greeson JM, et al. The many facets of mindfulness and the prediction of change following mindfulness-based stress reduction (MBSR). J Clin Psychol 2018;74:523–35. 10.1002/jclp.22521 28815600 PMC5815955

[40] Burton H, Sagoo GS, Pharoah PDP. Time to revisit Geoffrey rose: strategies for prevention in the genomic era? Italian Journal of Public Health 2012;9:e8665–1. 10.2427/8665

[41] Tierney JF, Fisher DJ, Burdett S, et al. Comparison of aggregate and individual participant data approaches to meta-analysis of randomised trials: an observational study. PLoS Med 2020;17:e1003019. 10.1371/journal.pmed.1003019 32004320 PMC6993967

[42] Shamseer L, Moher D, Clarke M, et al. Preferred reporting items for systematic review and meta-analysis protocols (PRISMA-P) 2015: elaboration and explanation. BMJ 2015;350:g7647. 10.1136/bmj.g7647 25555855

[43] Scottish Intercollegiate Guidelines Network. Search filters, 2018. Available: https://www.sign.ac.uk/what-we-do/methodology/search-filters/ [Accessed 1 April 2022].

[44] Mayo-Wilson E, Li T, Fusco N, et al. Cherry-picking by Trialists and meta-analysts can drive conclusions about intervention efficacy. J Clin Epidemiol 2017;91:95–110. 10.1016/j.jclinepi.2017.07.014 28842290

[45] Jayewardene WP, Lohrmann DK, Erbe RG, et al. Effects of preventive online mindfulness interventions on stress and mindfulness: a meta-analysis of randomized controlled trials. Prev Med Rep 2017;5:150–9. 10.1016/j.pmedr.2016.11.013 28050336 PMC5199155

[46] RoB2 Development Group. Revised Cochrane risk-of-bias tool for randomized trials (rob 2), 2018. Available: https://sites.google.com/site/riskofbiastool/welcome/rob-2-0-tool/current-version-of-rob-2?authuser=0 [Accessed 21 Sep 2018].

[47] Sterne JAC, Savović J, Page MJ, et al. Rob 2: a revised tool for assessing risk of bias in randomised trials. BMJ 2019;366:l4898. 10.1136/bmj.l4898 31462531

[48] Savovic J, Turner RM, Mawdsley D, et al. Association between Risk-of-Bias assessments and results of randomized trials in Cochrane reviews: the ROBES Meta-Epidemiologic study. Am J Epidemiol 2018;187:1113–22. 10.1093/aje/kwx344 29126260 PMC5928453

[49] Guyatt GH, Oxman AD, Vist GE, et al. Grad: an emerging consensus on rating quality of evidence and strength of recommendations. BMJ 2008;336:924–6. 10.1136/bmj.39489.470347.AD 18436948 PMC2335261

[50] Riley RD, Tierney JF, Stewart LA, eds. Individual Participant Data Meta-Analysis: A Handbook for Healthcare Research. Wiley, 2021.

[51] McKenzie JE, Herbison GP, Deeks JJ. Impact of analysing continuous outcomes using final values, change scores and analysis of covariance on the performance of meta-analytic methods: a simulation study. Res Synth Methods 2016;7:371–86. 10.1002/jrsm.1196 26715122 PMC5217094

[52] Mavridis D, Salanti G. A practical introduction to multivariate meta-analysis. Stat Methods Med Res 2013;22:133–58. 10.1177/0962280211432219 22275379

[53] Riley RD, Higgins JPT, Deeks JJ. Interpretation of random effects meta-analyses. BMJ 2011;342:d549. 10.1136/bmj.d549 21310794

[54] Debray TPA, Moons KGM, van Valkenhoef G, et al. Get real in individual participant data (IPD) meta-analysis: a review of the methodology. Res Synth Methods 2015;6:293–309. 10.1002/jrsm.1160 26287812 PMC5042043

[55] White IR, Royston P, Wood AM. Multiple imputation using chained equations: issues and guidance for practice. Stat Med 2011;30:377–99. 10.1002/sim.4067 21225900

[56] Riley RD, Debray TPA, Fisher D, et al. Individual participant data meta-analysis to examine interactions between treatment effect and participant-level covariates: statistical recommendations for conduct and planning. Stat Med 2020;39:2115–37. 10.1002/sim.8516 32350891 PMC7401032

[57] Fisher DJ, Carpenter JR, Morris TP, et al. Meta-analytical methods to identify who benefits most from treatments: daft, deluded, or deft approach? BMJ 2017;356:j573. 10.1136/bmj.j573 28258124 PMC5421441

[58] White IR, Kaptoge S, Royston P, et al. Meta-Analysis of non-linear exposure-outcome relationships using individual participant data: a comparison of two methods. Stat Med 2019;38:326–38. 10.1002/sim.7974 30284314 PMC6492097

[59] Gu J, Strauss C, Bond R, et al. How do mindfulness-based cognitive therapy and mindfulness-based stress reduction improve mental health and wellbeing? A systematic review and meta-analysis of mediation studies. Clin Psychol Rev 2015;37:1–12. 10.1016/j.cpr.2015.01.006 25689576

[60] Klatt MD, Buckworth J, Malarkey WB. Effects of low-dose mindfulness-based stress reduction (MBSR-ld) on working adults. Health Educ Behav 2009;36:601–14. 10.1177/1090198108317627 18469160

