# Supplementary material for: Mindfulness-based programmes for mental health promotion in adults in non-clinical settings: protocol of an individual participant data meta-analysis of randomised controlled trials
Source: BMJ Open. 2022 Apr 10;12(4):e058976. doi: 10.1136/bmjopen-2021-058976 (PMC9003609; doi:10.1136/bmjopen-2021-058976)
Supplement: Supplementary data [file bmjopen-2021-058976supp001.pdf]

Mindfulness-based programmes for mental health promotion in adults in non-clinical settings: protocol of an individual participant data meta-analysis of randomised controlled trials

Online Supplementary Material

Appendix 1 - Search strategy for EMBASE (Ovid interface)

S1 exp meditation/ or exp mindfulness/
S2 (mindfullness or mindfulness or meditat*).ab. or (mindfullness or mindfulness or meditat*).ti.
S3 1 or 2
S4 clinical trial/
S5 randomized controlled trial/
S6 controlled clinical trial/
S7 multicenter study/
S8 phase 3 clinical trial/
S9 phase 4 clinical trial/
S10 double blind procedure/
S11 placebo/ S12 exp randomization/
S13 (randomi?ed controlled trial$ or rct or (random$ adj2 allocat$) or single blind$ or double blind$ or ((treble or triple) adj blind$) or placebo$).tw.
S14 Prospective Study.mp. or prospective study/
S15 4 or 5 or 6 or 7 or 8 or 9 or 10 or 11 or 12 or 13 or 14
S16 3 and 15

Galante J, *et al. BMJ Open* 2022; 12:e058976. doi: 10.1136/bmjopen-2021-058976

Appendix 2 – Aggregate data extraction form

**Categories**	**Extracted information**
Study Identification	Sponsorship sources, conflicts of interest, country, study setting, corresponding authors, institution, emails, date recruitment started, and year first published.
Methods	Study design, conceptualisation of mindfulness, incentives for participants, number of participants (total randomised and per arm), and power calculation.
Population	Inclusion and exclusion criteria, type of participant, group differences, baseline characteristics
Interventions	Intervention name, mention of intervention manual, whether it was an adaptation of another intervention (rationale), intensity, mindfulness components (type, frequency and duration), non-mindfulness components (type, frequency and duration), home practice length and type, group size, any individual tailoring, any booster sessions or support after the end of the programme, adherence to intervention manual, intervention setting, teacher competence, teacher characteristics, response to intervention (attendance, satisfaction, reasons for missing sessions), and whether participants paid to do the course.
Outcomes	Outcome measure used, time points, group sizes, effect measures available and extracted effect sizes.

Appendix 3 – Multiple imputation plan details

All imputations will be done separately by trial. This is compatible with a two-stage meta-analysis where results from imputed data sets are combined by Rubin's rules to give a trial-specific estimate ready for meta-analysis.

All imputations will also be done separately by randomised group. This respects all possible treatment-by-covariate interactions. If the trial is a cluster-RCT, clustering will be ignored in the imputation but allowed for in the analysis.

For each trial, individuals with all missing outcomes will be dropped before imputation, and the remaining missing outcomes will be multiply imputed either item by item or as a total score. If for *most* (>50%) individuals most (>50%) of the questionnaire items are available, we will impute the items of that questionnaire *for all of the individuals* in the trial; if not, we will impute total scores. We will use predictive mean matching.

All outcomes will be included and imputed in the same model (multivariate imputation by chained equations, MICE) because outcomes will act as auxiliary variables for each other. All of the moderators will be included and imputed in the same model so that they contribute to imputing the outcomes, but observations with imputed moderators will be dropped for analysis. We will choose a number of imputations at least equal to the percentage of incomplete cases.

Galante J, *et al. BMJ Open* 2022; 12:e058976. doi: 10.1136/bmjopen-2021-058976
