# Supplementary material for: Mindfulness-based programmes for mental health promotion in adults in non-clinical settings: protocol of an individual participant data meta-analysis of randomised controlled trials
Source: BMJ Open. 2022 Apr 10;12(4):e058976. doi: 10.1136/bmjopen-2021-058976 (PMC9003609; doi:10.1136/bmjopen-2021-058976)
Supplement: Author's manuscript [file bmjopen-2021-058976.draft_revisions.pdf]

BMJ Open

BMJ Open is committed to open peer review. As part of this commitment we make the peer review history of every article we publish publicly available.

When an article is published we post the peer reviewers' comments and the authors' responses online. We also post the versions of the paper that were used during peer review. These are the versions that the peer review comments apply to.

The versions of the paper that follow are the versions that were submitted during the peer review process. They are not the versions of record or the final published versions. They should not be cited or distributed as the published version of this manuscript.

BMJ Open is an open access journal and the full, final, typeset and author-corrected version of record of the manuscript is available on our site with no access controls, subscription charges or pay-per-view fees (http://bmjopen.bmj.com).

If you have any questions on BMJ Open's open peer review process please email info.bmjopen@bmj.com

Mindfulness-based programmes for mental health promotion in adults in non-clinical settings: protocol of an individual participant data meta-analysis of randomised controlled trials

Journal:	*BMJ Open*
Manuscript ID	bmjopen-2021-058976
Article Type:	Protocol
Date Submitted by the Author:	03-Nov-2021

Complete List of Authors:	Galante, Julieta; University of Cambridge, Department of Psychiatry; second affiliation National Institute for Health Research (NIHR) Applied Research Collaboration East of England), Department of Psychiatry<br>Friedrich, Claire; University of Cambridge, Department of Psychiatry<br>Dalgleish, Tim; Medical Research Council Cognition and Brain Sciences Unit<br>White, Ian; University College London Faculty of Population Health Sciences, Clinical Trials & Methodology<br>Jones, Peter; University of Cambridge, Department of Psychiatry

Keywords:	MENTAL HEALTH, COMPLEMENTARY MEDICINE, PUBLIC HEALTH

SCHOLARONE™
Manuscripts

Mindfulness-based programmes for mental health promotion in adults in non-clinical settings: protocol of an individual participant data meta-analysis of randomised controlled trials

Authors:

1. Julieta Galante (**corresponding author**), Department of Psychiatry, University of Cambridge, Douglas House, 18b Trumpington Road, Cambridge, CB2 8AH, United Kingdom (second affiliation: National Institute for Health Research (NIHR) Applied Research Collaboration East of England). Email: mjg231@cam.ac.uk
2. Claire Friedrich, Department of Psychiatry, University of Cambridge, Cambridge, United Kingdom.
3. Tim Dalgleish, MRC Cognition and Brain Sciences Unit, University of Cambridge, Cambridge, United Kingdom (second affiliation: Cambridgeshire and Peterborough NHS Foundation Trust)
4. Ian R White, MRC Clinical Trials Unit, University College London, London, United Kingdom.
5. Peter B Jones, Department of Psychiatry, University of Cambridge, Cambridge, United Kingdom (second affiliation: NIHR Applied Research Collaboration East of England)

For peer review only

ABSTRACT

Introduction

With mental ill health listed as a top cause of global disease burden, there is an urgent need to prioritise mental health promotion programmes. Mindfulness–based programmes (MBPs) are being widely implemented to reduce stress in non-clinical settings. In a recent aggregate-level meta-analysis we found that, compared with no intervention, these MBPs reduce average psychological distress. However, heterogeneity between studies impedes generalisation of effects across every setting. Study-level effect modifiers were insufficient to reduce heterogeneity; studying individual–level effect modifiers is warranted. This requires individual participant data (IPD) and larger samples than those found in existing individual trials.

Methods and analysis

We propose an IPD meta–analysis. Our primary aim is to see if, and how, baseline psychological distress, gender, age, education, and dispositional mindfulness moderate the effect of MBPs on distress. We will search 13 databases for good-quality randomised controlled trials (RCTs) comparing in–person, expert–defined MBPs in non-clinical settings with passive controls. Two researchers will independently select, extract, and appraise trials using the revised Cochrane Risk–of–Bias Tool (RoB2). Anonymised IPD of eligible trials will be sought from authors, who will be invited to collaborate.

The primary outcome will be psychological distress measured using psychometrically-validated questionnaires at 1 to 6 months after programme completion. Pairwise random-effects two-stage IPD meta-analyses will be conducted. Moderator analyses will follow a “deft” approach. We will estimate subgroup-specific intervention effects. Secondary outcomes and sensitivity analyses are pre-specified. Multiple imputation strategies will be applied to missing data.

Ethics and dissemination

The findings will refine our knowledge on the effectiveness of MBPs and help improve the targeting of MBPs in non-clinical settings. They will be shared in accessible formats with a range of stakeholders. Public and professional stakeholders are being involved in the planning, conduct and dissemination of this project.

PROSPERO registration number CRD42020200117

For peer review only

STRENGTHS AND LIMITATIONS OF THIS STUDY

1. This is, to our knowledge, the first individual-participant-data (IPD) meta-analysis assessing the effectiveness of mindfulness-based programmes to reduce psychological distress among adults in non-clinical settings, and how it varies as a function of individual differences.

2. Preceded by a comprehensive systematic review, this IPD meta-analysis will have greater statistical power to detect effect modifiers than any of the individual trials.

3. This IPD meta-analysis can overcome some, but not all, of the existing trials' methodological shortcomings.

4. As a secondary-data analysis, this study depends on trial data being shared; this factor can limit the validity and generalisability of the findings.

5. The outcomes and effect modifiers that can be assessed are limited to those that the existing trials have measured, and how they have measured them.

For peer review only

INTRODUCTION

Common mental health disorders such as depression are among the top worldwide causes of morbidity, generating a very significant burden on societies [1]. The COVID-19 pandemic, a global natural stressor, is increasing this burden [2]. The last decade has seen an expansion of mental health prevention and promotion programmes in workplaces, educational establishments, and other community settings [3]. They usually target psychological distress, a concept encompassing a range of disturbing or unpleasant mental or emotional experiences which, if unaddressed, can result in mental and physical health disorders [4].

Frequently promoted as a universal tool to reduce stress [5], mindfulness-based programmes (MBPs) are among the most commonly implemented preventive activities [6]. In the United States of America, mindfulness training is present at 79% of medical schools [7], and offered by 22% of employers [8]. MBPs typically define mindfulness as “the awareness that emerges through paying attention on purpose, in the present moment, and nonjudgmentally to the unfolding of experience moment by moment” [9]. Their core elements are an emphasis on teaching mindfulness meditation and mindful activities, scientific approaches to managing health, suitability for delivery in public institutions across a range of settings and cultures, and class-based experiences of collective and individual inquiry with a qualified teacher in a participatory learning process [10].

We recently completed a systematic review and aggregate-level meta-analysis of randomised controlled trials (RCTs) assessing MBPs for mental health promotion in adults in non-clinical settings (from now on referred to as our previous review)[11]. We found that, compared with no intervention, MBPs of the included studies, on average, reduced psychological distress (our most measured and robust outcome). However, given the heterogeneity between studies, the findings did not support generalisation of MBP effects across every setting. We investigated study-level factors that could moderate the effect of the MBPs on psychological distress, such as programme characteristics or type of population being targeted, but these were not able to fully explain the seen heterogeneity. Participant-level effect modifiers, such as participants’ prior mental health, may be at play.

Individual-participant-data (IPD) meta-analyses are the only practical choice for exploring how MBP effectiveness varies as a function of individual differences [12-14]. In IPD meta-analyses, rather than extracting summary data from trial publications, the original individual-level trial data are sought directly from trial authors. Aggregate-level meta-analyses (the most common ones) may give misleading individual-level moderator results because of study-level confounding [14]. Conducting further RCTs to perform sub-group analyses is expensive and impractical due to the large sample sizes required, particularly to find small-to-moderate effect sizes in low-risk populations. It is notoriously difficult to identify genuine predictors of differential response from single trials, as there is high potential for type I and type II errors [14]. IPD meta-analyses can obtain results for specific sub-groups of participants across studies, and differential effects can be assessed across individuals, which can help reduce between-study heterogeneity[15]. Other advantages of this approach are that data can be checked and re-analysed, and missing data can be accounted for at the individual level [16]. Finally, they can act as a stimulus for international collaboration, debate and consensus, and form the basis for further data sharing and open research. A key limitation of IPD meta-analyses is that the outcomes and effect modifiers that can be assessed are limited to those that the existing trials have measured, and how they have measured them. IPD meta-analyses also depend on trial authors’ willingness to share data, and on how well the trials were conducted.

For peer review only

The role of individual differences in MBPs

Preliminary evidence strongly suggests that the effectiveness of MBPs vary as a function of individual differences [17]. There have been several calls to study MBP effect modification more extensively, and small sample sizes have frequently been cited as a limiting factor [12 13 18-21].

Individuals with worse mental health to begin with may be the most likely to benefit because there is more room to what can be learnt. There is evidence that MBPs targeted at stressed groups [11 22], those with anxiety or mood disorders [23], those with higher symptom severity [24 25], or those experiencing stressful times [26] have larger effects. An IPD meta-analysis of mindfulness-based cognitive therapy (MBCT) to prevent recurrent depression relapse found a significant relative reduction in effect with better baseline status [27]. Most findings thus suggest that higher baseline distress levels strengthen intervention effects, although some have found no evidence of interaction [28].

A meta-analysis of workplace MBPs found a significant moderating effect of gender on well-being and life satisfaction [29]. This finding adds to previous evidence suggesting that MBP effects on men are smaller than those on women [20 30 31]. It has been posited that women tend to internalise their distress more, which may make techniques such as mindfulness work more favourably, while an externalising coping style, more frequently associated with men, may limit the effectiveness of MBPs [20]. Others proposed that neuroticism and conscientiousness, personality factors more common among women, may amplify MBP effects [20 30 31]. Some studies exploring gender as an effect modifier, including the MBCT IPD meta-analysis, have found no moderating effects [22 27].

Meta-analytic evidence suggests that MBPs for children and adolescents [32] and university students [33], have larger effects than those for adults [11]. While some studies reported no moderating effects of age, one study found age to moderate intervention effects on levels of anxiety, with older adults reporting smaller reductions in anxiety over time compared to their younger counterparts [34]. At play may be cognitive and cultural factors that are intrinsic to age such as plasticity and curiosity, or confounders such as education (e.g. young people belong to university student samples). However, age was not an effect modifier in the MBCT IPD meta-analysis and other studies [27 35].

Education levels are known to moderate the effectiveness of some psychological interventions [36]. Concerns have been voiced that current MBPs may not be inclusive of diverse education backgrounds because of their language and cultural references [37]. A recent meta-analysis has reported significant moderating effects of level of education in workplace MBPs, finding a larger improvement in well-being among more highly educated participants [29]. However, education was not an effect modifier in the MBCT IPD meta-analysis [27].

Baseline levels of dispositional mindfulness, a multidimensional construct reflecting an individual's focus and quality of their attention [38], may moderate MBP effects, but the evidence is inconsistent and shows a complex picture [39]. For example, Shapiro et al. report that participants with higher trait mindfulness at baseline experienced greater and long-lasting improvements in well-being and distress [40], while Greeson et al. found that baseline dispositional mindfulness did not moderate the effect of an MBP on depressive symptoms [35]. A higher level of dispositional mindfulness may be needed to engage with MBPs, but this may also limit the amount that is to be learnt.

With the proliferation of mindfulness provision in recent times, understanding what works, for whom and in what circumstances becomes a pressing issue. This information is essential to tailor

For peer review only

interventions, maximising effectiveness, cost-effectiveness and ensuring intervention harm minimisation [41].

We plan to conduct a systematic review and individual participant data meta-analysis to answer the following main research question: Do selected participant-level characteristics moderate the effect of mindfulness-based programmes (MBPs) on psychological distress among adults in non-clinical settings, and if so, how do they do it? Our main aim is to see whether and how baseline psychological distress, gender, age, education, and dispositional mindfulness moderate the effect of MBPs on psychological distress compared with no intervention. Effect modifiers for this IPD meta-analysis have been selected based on existing theories and empirical evidence, and on availability as they are commonly reported among trials and are comparable across international samples. Exploring these potential effect modifiers with IPD will address current limitations and support our understanding of individual differences in response to MBPs [42].

METHODS AND ANALYSIS

This protocol follows PRISMA-P guidelines [43].

Study search and selection

The search will update that of our previous review [11]. Thirteen databases will be included: Allied and Complementary Medicine (AMED), Applied Social Sciences Index and Abstracts (ASSIA), the Cochrane Central Register of Controlled Trials (CENTRAL), the Cumulative Index to Nursing and Allied Health Literature (CINAHL), Education Resources Information Center (ERIC), Electronic Theses Online Service (EThOS), Excerpta Medica Database (EMBASE), Medical Literature Analysis and Retrieval System Online (MEDLINE), ProQuest, PsycINFO, Scopus, Web of Science and World Health Organization International Clinical Trials Registry Platform. Predefined search strategies using keywords and controlled vocabulary will be adapted and applied to each database. Where possible the search terms mindful and meditation will be combined with a pre-tested, sensitive filter for randomised controlled trials designed by the Scottish Intercollegiate Guidelines Network (2018), otherwise they will be combined with “randomize”, “RCT”, “random allocation” and “random assignment”. Search terms will be modified to include truncation, proximity indicators and wild cards. Additionally, when applicable, subject headings will be exploded. The database search strategy for EMBASE is available in Appendix 1 of the online supplementary materials as an example; all the strategies are also available in the publication of our previous review [11]. In addition to the electronic search, we will inspect the reference lists of identified RCTs and reviews. No language limitations will be included. Non-public sources of studies will not be used in the searches [44], but authors will be contacted to provide information as outlined herein.

The review inclusion criteria are presented in Table 1. These are similar but narrower in scope than our previous review in order to produce a more focused and better-quality analysis, and because it is infeasible for us to collect IPD from the 136 RCTs included in that review. Online MBPs were excluded because we believe they are different enough from in-person MBPs (e.g. typically not group-based, and fully or semi-automated) to merit their own separate analysis [45].

Trials included in our previous review and studies found through the search update will be assessed for inclusion in this IPD meta-analysis. Two researchers will independently review the titles and abstracts of all records retrieved by the search. If both reviewers agree that a record does not meet eligibility criteria, it will be excluded. The full text of all remaining records will be obtained, and the

For peer review only - http://bmjopen.bmj.com/site/about/guidelines.xhtml

same eligibility criteria will be applied to them by the two reviewers for a final selection. Disagreements will be decided via consensus between two senior team members (TD and PBJ) blind to trial results.

Data collection and processing

Two reviewers will independently extract study-level characteristics of newly-identified studies into extraction forms similar to those used in our previous review (Appendix 2 of the online supplementary materials). Authors of eligible studies will be invited to collaborate. Publication co-authorship, help with data preparation and transfer, and secure and confidential data management will be offered. If necessary, authors other than the correspondent author will be contacted. IPD will be considered unavailable where no authors have responded to multiple contact attempts, where authors indicate that they no longer have access to the data, or where authors decline to participate.

Anonymised trial IPD relevant to the analyses proposed herein will be requested from authors who accept our invitation. We will request IPD for all randomised participants, independently of whether trial publications used all of the data or only a fraction. We will prefer datasets without imputed missing data.

Participant-level data characteristics will be checked as follows using structured forms. IPD from each trial will be checked for missing participants (e.g. compare IPD samples against trial CONSORT diagrams to ensure that IPD from all randomised participants is included if available), for missing outcomes and missing pre-specified effect modifiers (compared against trial publications and protocols), and for invalid, out of range or inconsistent items (e.g. unusually old or young participants), before being converted to standard format. We will request individual items from questionnaires, recalculating scale-specific scores where possible. We will check with trial authors whether any questionnaire items had been reversed, if applicable. IPD will be cross-examined against the summary statistics reported in trial publications. Inconsistencies will be checked by another reviewer. If they confirm that the numbers do not match, we will attempt to explain the difference (e.g. the publication may have used a per-protocol sample and we may have used the full randomized sample), and we will contact trial authors for clarification until inconsistencies are understood, and corrected if applicable.

Risk of bias assessment

Two reviewers will independently assess newly-found trials' risk of bias using the revised Cochrane risk-of-bias tool (RoB2) for randomised trials applied to the outcomes included in this review [46 47]. This tool stringently measures potential bias across five sources: (1) randomisation, (2) deviations from intended interventions, (3) missing outcome data, (4) measurement of the outcome, and (5) selection of the reported result. We will resolve discrepancies through discussion, involving a third reviewer where necessary.

Our previous review has found that many trials have high risk of bias from several sources, reducing confidence in the cumulative evidence. To understand how results were affected, we performed a sensitivity analysis removing trials deemed to be at high risk of bias from three or more sources, which divided the sample into roughly equal parts. The sensitivity analysis showed that the results were generally sensitive to this criterion. Accordingly, to maximize confidence in the IPD meta-analysis results and to maintain consistency with our previous review, we plan to only include trials with a maximum of two high risk-of-bias domains, as assessed before obtaining IPD. These are the trials likely to provide the most reliable evidence in the field. We acknowledge, however, that this

For peer review only

criterion is sub-optimal. The RoB2 tool has not been validated as a scale, so there are no validated cut-off points and domains may not be interchangeable [48].

Once studies have been selected and IPD obtained, risk of bias for individual studies will be updated according to the IPD available (e.g. risk lowered if IPD includes participants missing in published trial reports). We will check allocation for any unusual patterns. When key aspects are unclear, we will seek information from study authors. In order to assess the confidence in the cumulative evidence, we will use the GRADE approach (Guyatt et al. 2008).

Effect measures

The main outcome will be self-reported psychological distress measured between one and six months after programme completion using psychometrically valid questionnaires scored on a continuous scale (e.g. Perceived Stress Scale, General Health Questionnaire, Depression, Anxiety and Stress Scale). Questionnaires asking about fleeting states (e.g. "How do you feel now?") will be excluded.

Post-intervention psychological distress measures (i.e. those taken less than one month after programme completion) will be grouped and considered as a secondary outcome: they do not inform stable changes, therefore are less useful for understanding the real-life impact of MBPs. Psychological distress follow-up measured beyond 6 months will be grouped and also considered as a secondary outcome. If a study measured the outcome more than once within the time point range of interest, the longest follow-up will be used.

We expect that trials will use different questionnaires to measure psychological distress, therefore we will standardise them using z-scores. . We will calculate the the ANCOVA estimate (final score adjusted for baseline score) and adjust it for the available pre-specified effect modifiers [49 50].

If a trial reports more than one psychological distress measure within the same time point range, we will prefer the one assigned as primary outcome by the trialists; if this is not stated or none are primary outcomes, the one with best psychometric properties; if they have similar properties, the one that is used most frequently in the other studies. Full questionnaire scales and untransformed data will be preferred.

Data synthesis

Although this project focuses on the effect moderator analyses, overall effects will be calculated and will be compared with those found in our previous review. Data synthesis will be quantitative.. Two-stage IPD meta-analyses will be performed, as they automatically stratify parameter estimates by trial, use well-known meta-analysis methods, are more transparent than one-stage methods, and easily enable forest plots [49]. . They will be univariate for the time-point ranges for which data from all the trials are available, otherwise they will be multivariate and include all available time-point ranges [51].

Stage one of the two-stage IPD meta-analyses will involve conducting linear regressions separately by trial to estimate the trial's intervention effects. The models will include the baseline measurement of the outcome and the pre-specified effect modifiers available for that trial [49]. Stage two will combine the intervention effects from each trial using pairwise random-effects meta-analyses (a common effect is highly implausible) within comparator categories.

For peer review only

The main analysis will compare MBPs with a combination of all the passive control groups. If the included trials also compared MBPs with other interventions, these will be grouped under the comparator 'active control', and effects will be explored for this comparison in secondary analyses. In the event of finding multi-armed trials with multiple control groups that fit one category, these control groups will be combined. Two-arm trials that compare two eligible MBPs with each other will not be included. In multi-arm trials that do this, the two MBP arms will be combined for meta-analysis.

Estimation of heterogeneity will be performed using restricted maximum likelihood. To quantify the heterogeneity in the intervention effect, approximate prediction intervals will be calculated [52]. Intention-to-treat analyses of individual trials will be conducted for verification, to compare against published analyses and to discuss reasons for potential differences. Trials for which IPD are not made available will be included in a sensitivity analysis incorporating the available aggregate data. Results will be compared with IPD-data-only results [16].

Multiple imputation strategies will be applied to missing data (details in Appendix 3 of the online supplementary materials) [53 49 54]. A sensitivity analysis will compare results of imputed datasets with observed datasets. We will assess departures of the data missing at random assumption in sensitivity analyses at the individual study level, modelling missing data as 10% and 20% worse psychological stress scores than observed data. We will also explore the scenarios of missing distress scores in the intervention arm being worse than passive control group scores. In the mindfulness group participants who felt worse may have been less willing to answer because they were expecting an improvement or thought that they had done something wrong. Instead, passive control group participants may have expected to feel worse. We will explore how much worse missing outcome scores in the mindfulness arms would need to be for the significance and direction of the intervention effect to change.

Moderator analyses

The main moderator analyses will look at the effect of the moderators of interest one by one; if multiple interaction effects are found we will explore multivariable options to adjust for confounding as a secondary analysis. For each of the main moderator analyses, a treatment by participant covariate interaction term will be incorporated in the intervention effect trial regression models (first stage of two-stage meta-analysis), and the estimated interactions will be combined in a random effects meta-analysis. This method, known as the "deft" approach, will account for clustering of participants and separate out within-study and across-study information, avoiding ecological bias [55 56].

We will estimate subgroup-specific intervention effects by repeating the analysis procedure with the interaction parameters fixed at their "deft" estimates. Trials and/or individuals with missing values on an effect modifier will be excluded from the estimation of that interaction. If we find interaction effects after confounding adjustment, we will present a predictive model. We will test whether there is evidence of non-linear effects; if we find such evidence we will explore nonlinear models [55 57].

Continuous variables will not be categorised for analysis. We expect that trials will use different questionnaires to measure baseline psychological distress and dispositional mindfulness; we will standardise them using z-scores. Education level data are usually collected in the form of categories with a natural ordering; if that is the case then a linear trend across categories will be assumed [56]. Where trials have used different categories for collecting education level data, we shall strive to retain an ordered-categorical approach where levels have been collapsed by, for instance, PhD=1,

For peer review only

BA=2, PhD/BA=1.5, or by estimating years of education. Genders other than man/woman will be combined into an "other" category.

ETHICS AND DISSEMINATION

No local ethics approval was deemed necessary for this project following consultation with the research governance team. Trial authors will be requested to anonymise data sets prior to sharing them, and asked to confirm they have obtained ethical approval for sharing trial data anonymously. Data management and analysis will take place at the Department of Psychiatry, University of Cambridge. Data as obtained from individual trial authors will be stored at the highly secure Clinical School Secure Data Hosting Service and checked for any residual identifiable data before making copies to be used in normal workstations.

Findings will be disseminated within the academic community through publication in high-impact journals, conference presentations and networking. Professional stakeholders will be reached through activities focused on discussing the applicability of the findings. Media channels, social media (@MSSatUoC), and a variety of presentation formats will be used to engage with the wider public.

PATIENT AND PUBLIC INVOLVEMENT

A public stakeholder group is providing input throughout the life of this project. Members bring experiential expertise on mindfulness' effects and how they interact with contextual or personal factors, and on mental health promotion in daily life. We train and support them so that they are able to conceptually understand the study and can co-produce it. Stakeholders shaped the research questions and prioritised outcomes and moderation analyses. They will be invited to contribute to the day-to-day research process as research partners, for example by selecting studies and extracting data. They will be involved in interpreting the results, creating an impact plan, and disseminating the findings. We are also involving a group of professional stakeholders.

ACKNOWLEDGEMENTS

We are extremely grateful to those in our professional and public stakeholder groups for their keen involvement.

AUTHORS' CONTRIBUTIONS

JG applied for research funding and is the guarantor. JG, PBJ, TD and IRW planned the study. JG and CF wrote the manuscript that was revised through discussion with all the authors. All authors read and approved the final manuscript.

FUNDING STATEMENT

This publication presents independent research funded by the National Institute for Health Research (NIHR). The views expressed are those of the authors and not necessarily those of the NHS, the NIHR or the Department of Health and Social Care. JG is funded by a NIHR Post-doctoral Fellowship for this

For peer review only

research project (salary and all project costs, PDF-2017-10-018, https://www.nihr.ac.uk/). CF's salary for this research project was funded by a Cambridgeshire & Peterborough NHS Foundation Trust grant awarded to JG (RNAG/552, https://www.cpft.nhs.uk/). IRW was supported by the UK Medical Research Council (MC_UU_12023/21, https://mrc.ukri.org/). TD was supported by the UK Medical Research Council (SUAG/043 G101400, https://mrc.ukri.org/), the Wellcome Trust (104908/Z/14/Z, 107496/Z/15/Z, https://wellcome.org/), and the NIHR Cambridge Biomedical Research Centre (RG85446, 247730, https://cambridgebrc.nihr.ac.uk/). PBJ is supported by the Wellcome Trust (095844/Z/11/Z, https://wellcome.org/), the UK Medical Research Council (MR/N019067/1, https://mrc.ukri.org/), and the NIHR ARC East of England (RNAG/564, https://arc-eoe.nihr.ac.uk/). The funders had no role in study design, data collection and analysis, decision to publish, or preparation of this protocol.

COMPETING INTERESTS STATEMENT

The authors have no conflicts of interest to declare.

For peer review only

REFERENCES

1. Vos T, Barber RM, Bell B, et al. Global, regional, and national incidence, prevalence, and years lived with disability for 301 acute and chronic diseases and injuries in 188 countries, 1990-2013: a systematic analysis for the Global Burden of Disease Study 2013. *The Lancet* 2015;386(9995):743-800. doi: 10.1016/S0140-6736(15)60692-4
2. Vindegaard N, Benros ME. COVID-19 pandemic and mental health consequences: Systematic review of the current evidence. *Brain Behav Immun* 2020;89:531-42. doi: 10.1016/j.bbi.2020.05.048 [published Online First: 2020/06/03]
3. Samele C. Increasing momentum in prevention of mental illness and mental health promotion across Europe. *BJPsych International* 2016;13(1):22-23.
4. Russ TC, Stamatakis E, Hamer M, et al. Association between psychological distress and mortality: individual participant pooled analysis of 10 prospective cohort studies. *BMJ (Clinical research ed)* 2012;345:e4933. doi: 10.1136/bmj.e4933 [published Online First: 2012/08/02]
5. Kabat-Zinn J. Foreword: Seeds of a necessary global renaissance in the making: the refining of psychology's understanding of the nature of mind, self, and embodiment through the lens of mindfulness and its origins at a key inflection point for the species. *Current opinion in psychology* 2019;28:xi-xvii. doi: 10.1016/j.copsyc.2019.02.005 [published Online First: 2019/05/18]
6. Burke A, Lam CN, Stussman B, et al. Prevalence and patterns of use of mantra, mindfulness and spiritual meditation among adults in the United States. *BMC complementary and alternative medicine* 2017;17(1):316. doi: 10.1186/s12906-017-1827-8 [published Online First: 2017/06/18]
7. Barnes N, Hattan P, Black DS, et al. An Examination of Mindfulness-Based Programs in US Medical Schools. *Mindfulness* 2017;8(2): 489–94. doi: 10.1007/s12671-016-0623-8
8. Fortune. Meditation Has Become A Billion-Dollar Business 2016 [Available from: http://fortune.com/2016/03/12/meditation-mindfulness-apps/ accessed 01/12/2016.
9. Kabat-Zinn J. Full Catastrophe Living, Revised Edition: How to cope with stress, pain and illness using mindfulness meditation. 2 ed. London: Piatkus 2013.
10. Crane RS, Brewer J, Feldman C, et al. What defines mindfulness-based programs? The warp and the weft. *Psychol Med* 2017;47(6):990-99. doi: 10.1017/S0033291716003317 [published Online First: 2016/12/30]
11. Galante J, Friedrich C, Dawson AF, et al. Mindfulness-based programmes for mental health promotion in adults in non-clinical settings: A systematic review and meta-analysis of randomised controlled trials. *PLOS Medicine* 2021;18(1):e1003481. doi: https://doi.org/10.1371/journal.pmed.1003481
12. Goldberg SB, Sun S, Davidson RJ. The empirical status of mindfulness based interventions: A systematic review of 44 meta-analyses of randomized controlled trials. *Perspectives on Psychological Science* 2020
13. De Vibe M. Mindfulness Based Stress Reduction (MBSR) for Improving Health, Quality of Life, and Social Functioning in Adults. *Campbell Systematic Reviews, DOI 104073/csr20123* 2012 doi: 10.4073/csr.2012.3
14. Hingorani AD, Windt DA, Riley RD, et al. Prognosis research strategy (PROGRESS) 4: stratified medicine research. *BMJ (Clinical research ed)* 2013;346:e5793. doi: 10.1136/bmj.e5793 [published Online First: 2013/02/07]
15. Ioannidis JPA, Lau J. Uncontrolled Pearls, Controlled Evidence, Meta-Analysis and the Individual Patient. *Journal of clinical epidemiology* 1998;51(8):709-11.
16. Riley RD, Lambert PC, Abo-Zaid G. Meta-analysis of individual participant data: rationale, conduct, and reporting. *BMJ (Clinical research ed)* 2010;340:c221. doi: 10.1136/bmj.c221 [published Online First: 2010/02/09]

For peer review only

17. Keng SL, Smoski MJ, Robins CJ. Effects of mindfulness on psychological health: a review of empirical studies. *Clinical psychology review* 2011;31(6):1041-56. doi: 10.1016/j.cpr.2011.04.006 [published Online First: 2011/08/02]
18. Davidson RJ. Mindfulness-Based Cognitive Therapy and the Prevention of Depressive Relapse: Measures, Mechanisms, and Mediators. *JAMA psychiatry* 2016;73(6):547-8. doi: 10.1001/jamapsychiatry.2016.0135 [published Online First: 2016/04/28]
19. Ospina MB, Bond K, Karkhaneh M, et al. Meditation practices for health: state of the research. *Evidence report/technology assessment* 2007(155):1-263. [published Online First: 2007/09/04]
20. Rojiani R, Santoyo JF, Rahrig H, et al. Women Benefit More Than Men in Response to College-based Meditation Training. *Frontiers in psychology* 2017;8 doi: 10.3389/fpsyg.2017.00551
21. Shapiro SL, Brown KW, Thoresen C, et al. The moderation of Mindfulness-based stress reduction effects by trait mindfulness: results from a randomized controlled trial. *Journal of clinical psychology* 2011;67(3):267-77. doi: 10.1002/jclp.20761 [published Online First: 2011/01/22]
22. Schellekens MPJ, van den Hurk DGM, Prins JB, et al. Mindfulness-based stress reduction added to care as usual for lung cancer patients and/or their partners: A multicentre randomized controlled trial. *Psycho-oncology* 2017;26(12):2118-26. doi: 10.1002/pon.4430
23. Hofmann SG, Sawyer AT, Witt AA, et al. The effect of mindfulness-based therapy on anxiety and depression: A meta-analytic review. *J Consult Clin Psychol* 2010;78(2):169-83. doi: 10.1037/a0018555 [published Online First: 2010/03/31]
24. Roos CR, Bowen S, Witkiewitz K. Baseline patterns of substance use disorder severity and depression and anxiety symptoms moderate the efficacy of mindfulness-based relapse prevention. *Journal of consulting and clinical psychology* 2017;85(11):1041-51. doi: 10.1037/ccp0000249
25. Khoury B, Lecomte T, Fortin G, et al. Mindfulness-based therapy: a comprehensive meta-analysis. *Clinical psychology review* 2013;33(6):763-71. doi: 10.1016/j.cpr.2013.05.005 [published Online First: 2013/06/26]
26. Galante J, Stochl J, Dufour G, et al. Effectiveness of providing university students with a mindfulness-based intervention to increase resilience to stress: one-year follow-up of a pragmatic randomised controlled trial. *Journal of Epidemiology and Community Health* 2020;75:151-60. doi: http://dx.doi.org/10.1136/jech-2020-214390
27. Kuyken W, Warren FC, Taylor RS, et al. Efficacy of Mindfulness-Based Cognitive Therapy in Prevention of Depressive Relapse: An Individual Patient Data Meta-analysis From Randomized Trials. *JAMA psychiatry* 2016;73(6):565-74. doi: 10.1001/jamapsychiatry.2016.0076 [published Online First: 2016/04/28]
28. Fiocco AJ, Mallya S, Farzaneh M, et al. Exploring the benefits of mindfulness training in healthy community-dwelling older adults: A randomized controlled study using a mixed methods approach. *Mindfulness* 2018;10(4):737-48. doi: 10.1007/s12671-018-1041-x
29. Vonderlin R, Biermann M, Bohus M, et al. Mindfulness-Based Programs in the Workplace: a Meta-Analysis of Randomized Controlled Trials. *Mindfulness* 2020;11:1579–98. doi: 10.1007/s12671-020-01328-3
30. Galante J, Dufour G, Vainre M, et al. A mindfulness-based intervention to increase resilience to stress in university students (the Mindful Student Study): a pragmatic randomised controlled trial. *The Lancet Public Health* 2018;3(2):e72-e81. doi: 10.1016/s2468-2667(17)30231-1
31. De Vibe M, Solhaug I, Tyssen R, et al. Mindfulness training for stress management: a randomised controlled study of medical and psychology students. *BMC Medical Education* 2013;13(1):107.
32. Dunning DL, Griffiths K, Kuyken W, et al. The Effects of Mindfulness-Based Interventions on Cognition and Mental Health in Children and Adolescents: A Meta-Analysis of Randomised Controlled Trials. *J Child Psychol Psychiatry* 2019;60(3):244–58.

For peer review only

33. Dawson AF, Brown WW, Anderson J, et al. Mindfulness-based Interventions for University Students: A Systematic Review and Meta-analysis of Randomized Controlled Trials. *Applied Psychology: Health and Well-Being* 2020;12(2):384-410.
34. Nyklicek I, Irrmischer M. For Whom Does Mindfulness-Based Stress Reduction Work? Moderating Effects of Personality. *Mindfulness (N Y)* 2017;8(4):1106-16. doi: 10.1007/s12671-017-0687-0 [published Online First: 2017/08/02]
35. Greeson JM, Smoski MJ, Suarez EC, et al. Decreased Symptoms of Depression After Mindfulness-Based Stress Reduction: Potential Moderating Effects of Religiosity, Spirituality, Trait Mindfulness, Sex, and Age. *The Journal of Alternative and Complementary Medicine* 2015;21(3):166-74. doi: 10.1089/acm.2014.0285
36. Ebert DD, Donkin L, Andersson G, et al. Does Internet-based guided-self-help for depression cause harm? An individual participant data meta-analysis on deterioration rates and its moderators in randomized controlled trials. *Psychological Medicine* 2016;46(13):2679-93. doi: 10.1017/s0033291716001562
37. Russell L, Ugalde A, White V, et al. Relevance of mindfulness practices for culturally and linguistically diverse cancer populations. *Psycho-oncology* 2019;28(11):2250-52. doi: 10.1002/pon.5221 [published Online First: 2019/09/06]
38. Rau HK, Williams PG. Dispositional mindfulness: A critical review of construct validation research. *Personality and Individual Differences* 2016;93:32-43. doi: 10.1016/j.paid.2015.09.035
39. Gawrysiak MJ, Grassetti SN, Greeson JM, et al. The many facets of mindfulness and the prediction of change following mindfulness-based stress reduction (MBSR). *Journal of clinical psychology* 2018;74(4):523-35. doi: 10.1002/jclp.22521 [published Online First: 2017/08/18]
40. Shapiro SL, Brown KW, Thoresen C, et al. The moderation of Mindfulness-based stress reduction effects by trait mindfulness: Results from a randomized controlled trial. *Journal of clinical psychology* 2011;67(3):267-77. doi: https://doi.org/10.1002/jclp.20761
41. Burton H, Sagoo GS, Pharoah PDP, et al. Time to revisit Geoffrey Rose: strategies for prevention in the genomic era? *Italian Journal of Public Health* 2012;9(4):e8665-1. doi: 10.2427/8665
42. Tierney JF, Fisher DJ, Burdett S, et al. Comparison of aggregate and individual participant data approaches to meta-analysis of randomised trials: An observational study. *PLoS Med* 2020;17(1):e1003019. doi: 10.1371/journal.pmed.1003019 [published Online First: 2020/02/01]
43. Shamseer L, Moher D, Clarke M, et al. Preferred reporting items for systematic review and meta-analysis protocols (PRISMA-P) 2015: elaboration and explanation. *BMJ (Clinical research ed)* 2015;350:g7647. doi: 10.1136/bmj.g7647 [published Online First: 2015/01/04]
44. Mayo-Wilson E, Li T, Fusco N, et al. Cherry-picking by trialists and meta-analysts can drive conclusions about intervention efficacy. *Journal of clinical epidemiology* 2017;91:95-110. doi: 10.1016/j.jclinepi.2017.07.014 [published Online First: 2017/08/27]
45. Jayawardene WP, Lohrmann DK, Erbe RG, et al. Effects of preventive online mindfulness interventions on stress and mindfulness: A meta-analysis of randomized controlled trials. *Preventive Medicine Reports* 2017;5:150-59. doi: 10.1016/j.pmedr.2016.11.013
46. RoB2 Development Group. Revised Cochrane risk-of-bias tool for randomized trials (RoB 2) 2018 [updated 11 September 2018. Available from: https://sites.google.com/site/riskofbiastool/welcome/rob-2-0-tool/current-version-of-rob-2?authuser=0 accessed 21 September 2018
47. Sterne JAC, Savović J, Page MJ, et al. RoB 2: a revised tool for assessing risk of bias in randomised trials. *BMJ (Clinical research ed)* 2019;366:l4898. doi: 10.1136/bmj.l4898
48. Savovic J, Turner RM, Mawdsley D, et al. Association Between Risk-of-Bias Assessments and Results of Randomized Trials in Cochrane Reviews: The ROBES Meta-Epidemiologic Study. *American journal of epidemiology* 2018;187(5):1113-22. doi: 10.1093/aje/kwx344
49. Riley RD, Tierney JF, Stewart LA, editors. *Individual Participant Data Meta-Analysis: A Handbook for Healthcare Research*: Wiley, 2021.

For peer review only

50. McKenzie JE, Herbison GP, Deeks JJ. Impact of analysing continuous outcomes using final values, change scores and analysis of covariance on the performance of meta-analytic methods: a simulation study. *Research synthesis methods* 2016;7(4):371-86. doi: 10.1002/jrsm.1196 [published Online First: 2015/12/31]
51. Mavridis D, Salanti G. A practical introduction to multivariate meta-analysis. *Stat Methods Med Res* 2013;22(2):133-58. doi: 10.1177/0962280211432219 [published Online First: 2012/01/26]
52. Riley RD, Higgins JPT, Deeks JJ. Interpretation of random effects meta-analyses. *BMJ (Clinical research ed)* 2011;342:d549.
53. Debray TP, Moons KG, van Valkenhoef G, et al. Get real in individual participant data (IPD) meta-analysis: a review of the methodology. *Research synthesis methods* 2015;6(4):293-309. doi: 10.1002/jrsm.1160 [published Online First: 2015/08/20]
54. White IR, Royston P, Wood AM. Multiple imputation using chained equations: Issues and guidance for practice. *Statistics in medicine* 2011;30(4):377-99. doi: 10.1002/sim.4067 [published Online First: 2011/01/13]
55. Riley RD, Debray TPA, Fisher D, et al. Individual participant data meta-analysis to examine interactions between treatment effect and participant-level covariates: Statistical recommendations for conduct and planning. *Statistics in medicine* 2020;39(15):2115-37. doi: 10.1002/sim.8516 [published Online First: 2020/05/01]
56. Fisher DJ, Carpenter JR, Morris TP, et al. Meta-analytical methods to identify who benefits most from treatments: daft, deluded, or deft approach? *BMJ (Clinical research ed)* 2017;356:j573. doi: 10.1136/bmj.j573 [published Online First: 2017/03/05]
57. White IR, Kaptoge S, Royston P, et al. Meta-analysis of non-linear exposure-outcome relationships using individual participant data: A comparison of two methods. *Statistics in medicine* 2019;38(3):326-38. doi: 10.1002/sim.7974 [published Online First: 2018/10/05]
58. Gu J, Strauss C, Bond R, et al. How do mindfulness-based cognitive therapy and mindfulness-based stress reduction improve mental health and wellbeing? A systematic review and meta-analysis of mediation studies. *Clinical psychology review* 2015;37C:1-12. doi: 10.1016/j.cpr.2015.01.006 [published Online First: 2015/02/18]
59. Klatt MD, Buckworth J, Malarkey WB. Effects of low-dose mindfulness-based stress reduction (MBSR-ld) on working adults. *Health Education and Behavior* 2009;36(3):601-14. doi: http://dx.doi.org/10.1177/1090198108317627

For peer review only

TABLES

TABLE 1. REVIEW INCLUSION CRITERIA

Study aspect	Inclusion criterion
Design	Parallel-arm randomised controlled trials (RCTs) including cluster RCTs
Intervention	Group-based first generation MBPs as defined by Crane et al [10], with a minimum intensity of four one-hour in-person teacher-led sessions or equivalent*.
Comparison	Passive control groups such as no intervention, waitlists, or treatment-as-usual if the MBP arm also had access to it.
Population	Adult (18+ year old) participants living in the community, as long as the trial had not selected them for having any particular clinical condition. MBPs targeting specific community groups were included. Trials with slightly younger participants (e.g. those in university settings where some students will turn 18 during the first academic year) will be included.
Outcomes	Self-reported psychological distress measured between one and six months after MBP completion.
Effect modifiers	At least one of the following has been measured: baseline psychological distress, gender, age, education, and dispositional mindfulness.
Quality	A maximum of two high risk-of-bias sources as assessed using the RoB2 tool [47] before obtaining IPD (rationale in "Risk of Bias Assessment" section)

* Four MBP sessions were used as the "minimum dose" for participants in previous studies [58], and one-hour sessions are common in non-clinical settings [59]

For peer review only

Mindfulness-based programmes for mental health promotion in adults in non-clinical settings: protocol of an individual participant data meta-analysis of randomised controlled trials

Online Supplementary Material

Appendix 1 - Search strategy for EMBASE (Ovid interface)

S1 exp meditation/ or exp mindfulness/
S2 (mindfullness or mindfulness or meditat*).ab. or (mindfullness or mindfulness or meditat*).ti.
S3 1 or 2
S4 clinical trial/
S5 randomized controlled trial/
S6 controlled clinical trial/
S7 multicenter study/
S8 phase 3 clinical trial/
S9 phase 4 clinical trial/
S10 double blind procedure/
S11 placebo/ S12 exp randomization/
S13 (randomi?ed controlled trial$ or rct or (random$ adj2 allocat$) or single blind$ or double blind$ or ((treble or triple) adj blind$) or placebo$).tw.
S14 Prospective Study.mp. or prospective study/
S15 4 or 5 or 6 or 7 or 8 or 9 or 10 or 11 or 12 or 13 or 14
S16 3 and 15

For peer review only

Appendix 2 – Aggregate data extraction form

**Categories**	**Extracted information**
Study Identification	Sponsorship sources, conflicts of interest, country, study setting, corresponding authors, institution, emails, date recruitment started, and year first published.
Methods	Study design, conceptualisation of mindfulness, incentives for participants, number of participants (total randomised and per arm), and power calculation.
Population	Inclusion and exclusion criteria, type of participant, group differences, baseline characteristics
Interventions	Intervention name, mention of intervention manual, whether it was an adaptation of another intervention (rationale), intensity, mindfulness components (type, frequency and duration), non-mindfulness components (type, frequency and duration), home practice length and type, group size, any individual tailoring, any booster sessions or support after the end of the programme, adherence to intervention manual, intervention setting, teacher competence, teacher characteristics, response to intervention (attendance, satisfaction, reasons for missing sessions), and whether participants paid to do the course.
Outcomes	Outcome measure used, time points, group sizes, effect measures available and extracted effect sizes.

For peer review only

Appendix 3 – Multiple imputation plan details

All imputations will be done separately by trial. This is compatible with a two-stage meta-analysis where results from imputed data sets are combined by Rubin's rules to give a trial-specific estimate ready for meta-analysis.

All imputations will also be done separately by randomised group. This respects all possible treatment-by-covariate interactions. If the trial is a cluster-RCT, clustering will be ignored in the imputation but allowed for in the analysis.

For each trial, individuals with all missing outcomes will be dropped before imputation, and the remaining missing outcomes will be multiply imputed either item by item or as a total score. If for *most* (>50%) individuals most (>50%) of the questionnaire items are available, we will impute the items of that questionnaire *for all of the individuals* in the trial; if not, we will impute total scores. We will use predictive mean matching.

All outcomes will be included and imputed in the same model (multivariate imputation by chained equations, MICE) because outcomes will act as auxiliary variables for each other. All of the moderators will be included and imputed in the same model so that they contribute to imputing the outcomes, but observations with imputed moderators will be dropped for analysis. We will choose a number of imputations at least equal to the percentage of incomplete cases.

For peer review only

PRISMA-P (Preferred Reporting Items for Systematic review and Meta-Analysis Protocols) 2015 checklist: recommended items to address in a systematic review protocol*

Section and topic	Item No	Checklist item	Reported
**ADMINISTRATIVE INFORMATION**			
**Title:**			
Identification	1a	Identify the report as a protocol of a systematic review	✓
Update	1b	If the protocol is for an update of a previous systematic review, identify as such	✓
**Registration**	2	If registered, provide the name of the registry (such as PROSPERO) and registration number	✓
**Authors:**			
Contact	3a	Provide name, institutional affiliation, e-mail address of all protocol authors; provide physical mailing address of corresponding author	✓
Contributions	3b	Describe contributions of protocol authors and identify the guarantor of the review	✓
**Amendments**	4	If the protocol represents an amendment of a previously completed or published protocol, identify as such and list changes; otherwise, state plan for documenting important protocol amendments	✓
**Support:**			
Sources	5a	Indicate sources of financial or other support for the review	✓
Sponsor	5b	Provide name for the review funder and/or sponsor	✓
Role of sponsor or funder	5c	Describe roles of funder(s), sponsor(s), and/or institution(s), if any, in developing the protocol	✓
**INTRODUCTION**			
**Rationale**	6	Describe the rationale for the review in the context of what is already known	✓
**Objectives**	7	Provide an explicit statement of the question(s) the review will address with reference to participants, interventions, comparators, and outcomes (PICO)	✓
**METHODS**			
**Eligibility criteria**	8	Specify the study characteristics (such as PICO, study design, setting, time frame) and report characteristics (such as years considered, language, publication status) to be used as criteria for eligibility for the review	✓
**Information sources**	9	Describe all intended information sources (such as electronic databases, contact with study authors, trial registers or other grey literature sources) with planned dates of coverage	✓

For peer review only

**Search strategy**	10	Present draft of search strategy to be used for at least one electronic database, including planned limits, such that it could be repeated	✓
**Study records:**			
Data management	11a	Describe the mechanism(s) that will be used to manage records and data throughout the review	✓
Selection process	11b	State the process that will be used for selecting studies (such as two independent reviewers) through each phase of the review (that is, screening, eligibility and inclusion in meta-analysis)	✓
Data collection process	11c	Describe planned method of extracting data from reports (such as piloting forms, done independently, in duplicate), any processes for obtaining and confirming data from investigators	✓
Data items	12	List and define all variables for which data will be sought (such as PICO items, funding sources), any pre-planned data assumptions and simplifications	✓
**Outcomes and prioritization**	13	List and define all outcomes for which data will be sought, including prioritization of main and additional outcomes, with rationale	✓
**Risk of bias in individual studies**	14	Describe anticipated methods for assessing risk of bias of individual studies, including whether this will be done at the outcome or study level, or both; state how this information will be used in data synthesis	✓
**Data synthesis**	15a	Describe criteria under which study data will be quantitatively synthesised	✓
	15b	If data are appropriate for quantitative synthesis, describe planned summary measures, methods of handling data and methods of combining data from studies, including any planned exploration of consistency (such as I^2, Kendall's τ)	✓
	15c	Describe any proposed additional analyses (such as sensitivity or subgroup analyses, meta-regression)	✓
	15d	If quantitative synthesis is not appropriate, describe the type of summary planned	✓
**Meta-bias(es)**	16	Specify any planned assessment of meta-bias(es) (such as publication bias across studies, selective reporting within studies)	✓
**Confidence in cumulative evidence**	17	Describe how the strength of the body of evidence will be assessed (such as GRADE)	✓

From: Shamseer L, Moher D, Clarke M, Ghersi D, Liberati A, Petticrew M, Shekelle P, Stewart L, PRISMA-P Group. Preferred reporting items for systematic review and meta-analysis protocols (PRISMA-P) 2015: elaboration and explanation. BMJ. 2015 Jan 2;349(jan02 1):g7647.

For peer review only

BMJ Open

Mindfulness-based programmes for mental health promotion in adults in non-clinical settings: protocol of an individual participant data meta-analysis of randomised controlled trials

Journal:	*BMJ Open*
Manuscript ID	bmjopen-2021-058976.R1
Article Type:	Protocol
Date Submitted by the Author:	07-Feb-2022
Complete List of Authors:	Galante, Julieta; University of Cambridge, Department of Psychiatry; second affiliation National Institute for Health Research (NIHR) Applied Research Collaboration East of England), Department of Psychiatry<br>Friedrich, Claire; University of Cambridge, Department of Psychiatry<br>Dalgleish, Tim; Medical Research Council Cognition and Brain Sciences Unit<br>White, Ian; University College London Faculty of Population Health Sciences, Clinical Trials & Methodology<br>Jones, Peter; University of Cambridge, Department of Psychiatry
**Primary Subject Heading**:	Mental health
Secondary Subject Heading:	Public health
Keywords:	MENTAL HEALTH, COMPLEMENTARY MEDICINE, PUBLIC HEALTH

SCHOLARONE™
Manuscripts

Mindfulness-based programmes for mental health promotion in adults in non-clinical settings: protocol of an individual participant data meta-analysis of randomised controlled trials

Authors:

1. Julieta Galante (**corresponding author**), Department of Psychiatry, University of Cambridge, Douglas House, 18b Trumpington Road, Cambridge, CB2 8AH, United Kingdom (second affiliation: National Institute for Health Research (NIHR) Applied Research Collaboration East of England). Email: mjg231@cam.ac.uk
2. Claire Friedrich, Department of Psychiatry, University of Cambridge, Cambridge, United Kingdom. cf510@medschl.cam.ac.uk
3. Tim Dalgleish, MRC Cognition and Brain Sciences Unit, University of Cambridge, Cambridge, United Kingdom (second affiliation: Cambridgeshire and Peterborough NHS Foundation Trust). Tim.Dalgleish@mrc-cbu.cam.ac.uk
4. Ian R White, MRC Clinical Trials Unit, University College London, London, United Kingdom. ian.white@ucl.ac.uk
5. Peter B Jones, Department of Psychiatry, University of Cambridge, Cambridge, United Kingdom (second affiliation: NIHR Applied Research Collaboration East of England). pbj21@cam.ac.uk

For peer review only

For peer review only - http://bmjopen.bmj.com/site/about/guidelines.xhtml

ABSTRACT

Introduction

With mental ill health listed as a top cause of global disease burden, there is an urgent need to prioritise mental health promotion programmes. Mindfulness–based programmes (MBPs) are being widely implemented to reduce stress in non-clinical settings. In a recent aggregate-level meta-analysis we found that, compared with no intervention, these MBPs reduce average psychological distress. However, heterogeneity between studies impedes generalisation of effects across every setting. Study-level effect modifiers were insufficient to reduce heterogeneity; studying individual–level effect modifiers is warranted. This requires individual participant data (IPD) and larger samples than those found in existing individual trials.

Methods and analysis

We propose an IPD meta–analysis. Our primary aim is to see if, and how, baseline psychological distress, gender, age, education, and dispositional mindfulness moderate the effect of MBPs on distress. We will search 13 databases for good-quality randomised controlled trials (RCTs) comparing in–person, expert–defined MBPs in non-clinical settings with passive controls. Two researchers will independently select, extract, and appraise trials using the revised Cochrane Risk–of–Bias Tool (RoB2). Anonymised IPD of eligible trials will be sought from authors, who will be invited to collaborate.

The primary outcome will be psychological distress measured using psychometrically-validated questionnaires at 1 to 6 months after programme completion. Pairwise random-effects two-stage IPD meta-analyses will be conducted. Moderator analyses will follow a "deft" approach. We will estimate subgroup-specific intervention effects. Secondary outcomes and sensitivity analyses are pre-specified. Multiple imputation strategies will be applied to missing data.

Ethics and dissemination

The findings will refine our knowledge on the effectiveness of MBPs and help improve the targeting of MBPs in non-clinical settings. They will be shared in accessible formats with a range of stakeholders. Public and professional stakeholders are being involved in the planning, conduct and dissemination of this project.

PROSPERO registration number CRD42020200117

For peer review only - http://bmjopen.bmj.com/site/about/guidelines.xhtml

STRENGTHS AND LIMITATIONS OF THIS STUDY

1. This is, to our knowledge, the first individual-participant-data (IPD) meta-analysis assessing the effectiveness of mindfulness-based programmes to reduce psychological distress among adults in non-clinical settings, and how it varies as a function of individual differences.

2. Preceded by a comprehensive systematic review, this IPD meta-analysis will have greater statistical power to detect effect modifiers than any of the individual trials.

3. This IPD meta-analysis can overcome some, but not all, of the existing trials' methodological shortcomings.

4. As a secondary-data analysis, this study depends on trial data being shared; this factor can limit the validity and generalisability of the findings.

5. The outcomes and effect modifiers that can be assessed are limited to those that the existing trials have measured, and how they have measured them.

For peer review only

For peer review only - http://bmjopen.bmj.com/site/about/guidelines.xhtml

INTRODUCTION

Common mental health disorders such as depression are among the top worldwide causes of morbidity, generating a very significant burden on societies [1]. The COVID-19 pandemic, a global natural stressor, is increasing this burden [2]. The last decade has seen an expansion of mental health prevention and promotion programmes in workplaces, educational establishments, and other community settings [3]. They usually target psychological distress, a concept encompassing a range of disturbing or unpleasant mental or emotional experiences which, if unaddressed, can result in mental and physical health disorders [4].

Frequently promoted as a universal tool to reduce stress [5], mindfulness-based programmes (MBPs) are among the most commonly implemented preventive activities [6]. In the United States of America, mindfulness training is present at 79% of medical schools [7], and offered by 22% of employers [8]. MBPs typically define mindfulness as "the awareness that emerges through paying attention on purpose, in the present moment, and nonjudgmentally to the unfolding of experience moment by moment" [9]. Their core elements are an emphasis on teaching mindfulness meditation and mindful activities, scientific approaches to managing health, suitability for delivery in public institutions across a range of settings and cultures, and class-based experiences of collective and individual inquiry with a qualified teacher in a participatory learning process [10].

We recently completed a systematic review and aggregate-level meta-analysis of randomised controlled trials (RCTs) assessing MBPs for mental health promotion in adults in non-clinical settings (from now on referred to as our previous review)[11]. We found that, compared with no intervention, MBPs of the included studies, on average, reduced psychological distress (our most measured and robust outcome). However, given the heterogeneity between studies, the findings did not support generalisation of MBP effects across every setting. We investigated study-level factors that could moderate the effect of the MBPs on psychological distress, such as programme characteristics or type of population being targeted, but these were not able to fully explain the seen heterogeneity. Participant-level effect modifiers, such as participants' prior mental health, may be at play.

Individual-participant-data (IPD) meta-analyses are the only practical choice for exploring how MBP effectiveness varies as a function of individual differences [12-14]. In IPD meta-analyses, rather than extracting summary data from trial publications, the original individual-level trial data are sought directly from trial authors. Aggregate-level meta-analyses (the most common ones) may give misleading individual-level moderator results because of study-level confounding [14]. Conducting further RCTs to perform sub-group analyses is expensive and impractical due to the large sample sizes required, particularly to find small-to-moderate effect sizes in low-risk populations. It is notoriously difficult to identify genuine predictors of differential response from single trials, as there is high potential for type I and type II errors [14]. IPD meta-analyses can obtain results for specific sub-groups of participants across studies, and differential effects can be assessed across individuals, which can help reduce between-study heterogeneity[15]. Other advantages of this approach are that data can be checked and re-analysed, and missing data can be accounted for at the individual level [16]. Finally, they can act as a stimulus for international collaboration, debate and consensus, and form the basis for further data sharing and open research. A key limitation of IPD meta-analyses is that the outcomes and effect modifiers that can be assessed are limited to those that the existing trials have measured, and how they have measured them. IPD meta-analyses also depend on trial authors' willingness to share data, and on how well the trials were conducted.

For peer review only - http://bmjopen.bmj.com/site/about/guidelines.xhtml

The role of individual differences in MBPs

Preliminary evidence strongly suggests that the effectiveness of MBPs vary as a function of individual differences [17]. There have been several calls to study MBP effect modification more extensively, and small sample sizes have frequently been cited as a limiting factor [12 13 18-21].

Individuals with worse mental health to begin with may be the most likely to benefit because there is more room to what can be learnt. There is evidence that MBPs targeted at stressed groups [11 22], those with anxiety or mood disorders [23], those with higher symptom severity [24 25], or those experiencing stressful times [26] have larger effects. An IPD meta-analysis of mindfulness-based cognitive therapy (MBCT) to prevent recurrent depression relapse found a significant relative reduction in effect with better baseline status [27]. Most findings thus suggest that higher baseline distress levels strengthen intervention effects, although some have found no evidence of interaction [28].

A meta-analysis of workplace MBPs found a significant moderating effect of gender on well-being and life satisfaction [29]. This finding adds to previous evidence suggesting that MBP effects on men are smaller than those on women [20 30 31]. It has been posited that women tend to internalise their distress more, which may make techniques such as mindfulness work more favourably, while an externalising coping style, more frequently associated with men, may limit the effectiveness of MBPs [20]. Others proposed that neuroticism and conscientiousness, personality factors more common among women, may amplify MBP effects [20 30 31]. Some studies exploring gender as an effect modifier, including the MBCT IPD meta-analysis, have found no moderating effects [22 27].

Meta-analytic evidence suggests that MBPs for children and adolescents [32] and university students [33], have larger effects than those for adults [11]. While some studies reported no moderating effects of age, one study found age to moderate intervention effects on levels of anxiety, with older adults reporting smaller reductions in anxiety over time compared to their younger counterparts [34]. At play may be cognitive and cultural factors that are intrinsic to age such as plasticity and curiosity, or confounders such as education (e.g. young people belong to university student samples). However, age was not an effect modifier in the MBCT IPD meta-analysis and other studies [27 35].

Education levels are known to moderate the effectiveness of some psychological interventions [36]. Concerns have been voiced that current MBPs may not be inclusive of diverse education backgrounds because of their language and cultural references [37]. A recent meta-analysis has reported significant moderating effects of level of education in workplace MBPs, finding a larger improvement in well-being among more highly educated participants [29]. However, education was not an effect modifier in the MBCT IPD meta-analysis [27].

Baseline levels of dispositional mindfulness, a multidimensional construct reflecting an individual's focus and quality of their attention [38], may moderate MBP effects, but the evidence is inconsistent and shows a complex picture [39]. For example, Shapiro et al. report that participants with higher trait mindfulness at baseline experienced greater and long-lasting improvements in well-being and distress [40], while Greeson et al. found that baseline dispositional mindfulness did not moderate the effect of an MBP on depressive symptoms [35]. A higher level of dispositional mindfulness may be needed to engage with MBPs, but this may also limit the amount that is to be learnt.

With the proliferation of mindfulness provision in recent times, understanding what works, for whom and in what circumstances becomes a pressing issue. This information is essential to tailor

For peer review only - http://bmjopen.bmj.com/site/about/guidelines.xhtml

interventions, maximising effectiveness, cost-effectiveness and ensuring intervention harm minimisation [41].

We plan to conduct a systematic review and individual participant data meta-analysis to answer the following main research question: Do selected participant-level characteristics moderate the effect of mindfulness-based programmes (MBPs) on psychological distress among adults in non-clinical settings, and if so, how do they do it? Our main aim is to see whether and how baseline psychological distress, gender, age, education, and dispositional mindfulness moderate the effect of MBPs on psychological distress compared with no intervention. Effect modifiers for this IPD meta-analysis have been selected based on existing theories and empirical evidence, and on availability as they are commonly reported among trials and are comparable across international samples. Exploring these potential effect modifiers with IPD will address current limitations and support our understanding of individual differences in response to MBPs [42].

For peer review only

METHODS AND ANALYSIS

This protocol follows PRISMA-P guidelines [43]. It has been publicly registered (PROSPERO registration number CRD42020200117).

Study search and selection

The search will update that of our previous review [11]. Thirteen databases will be included: Allied and Complementary Medicine (AMED), Applied Social Sciences Index and Abstracts (ASSIA), the Cochrane Central Register of Controlled Trials (CENTRAL), the Cumulative Index to Nursing and Allied Health Literature (CINAHL), Education Resources Information Center (ERIC), Electronic Theses Online Service (EThOS), Excerpta Medica Database (EMBASE), Medical Literature Analysis and Retrieval System Online (MEDLINE), ProQuest, PsycINFO, Scopus, Web of Science and World Health Organization International Clinical Trials Registry Platform. Predefined search strategies using keywords and controlled vocabulary will be adapted and applied to each database. Where possible the search terms mindful and meditation will be combined with a pre-tested, sensitive filter for randomised controlled trials designed by the Scottish Intercollegiate Guidelines Network (2018), otherwise they will be combined with “randomize”, “RCT”, “random allocation” and “random assignment”. Search terms will be modified to include truncation, proximity indicators and wild cards. Additionally, when applicable, subject headings will be exploded. The database search strategy for EMBASE is available in Appendix 1 of the online supplementary materials as an example; all the strategies are also available in the publication of our previous review [11]. In addition to the electronic search, we will inspect the reference lists of identified RCTs and reviews. No language limitations will be included. Non-public sources of studies will not be used in the searches [44], but authors will be contacted to provide information as outlined herein.

The review inclusion criteria are presented in Table 1. These are similar but narrower in scope than our previous review in order to produce a more focused and better-quality analysis, and because it is infeasible for us to collect IPD from the 136 RCTs included in that review. Online MBPs were excluded because we believe they are different enough from in-person MBPs (e.g. typically not group-based, and fully or semi-automated) to merit their own separate analysis [45].

Trials included in our previous review and studies found through the search update will be assessed for inclusion in this IPD meta-analysis. Two researchers will independently review the titles and abstracts of all records retrieved by the search. If both reviewers agree that a record does not meet

For peer review only - http://bmjopen.bmj.com/site/about/guidelines.xhtml

eligibility criteria, it will be excluded. The full text of all remaining records will be obtained, and the same eligibility criteria will be applied to them by the two reviewers for a final selection. Disagreements will be decided via consensus between two senior team members (TD and PBJ) blind to trial results.

Data collection and processing

Two reviewers will independently extract study-level characteristics of newly-identified studies into extraction forms similar to those used in our previous review (Appendix 2 of the online supplementary materials). Authors of eligible studies will be invited to collaborate. Publication co-authorship, help with data preparation and transfer, and secure and confidential data management will be offered. If necessary, authors other than the correspondent author will be contacted. IPD will be considered unavailable where no authors have responded to multiple contact attempts, where authors indicate that they no longer have access to the data, or where authors decline to participate.

Anonymised trial IPD relevant to the analyses proposed herein will be requested from authors who accept our invitation. We will request IPD for all randomised participants, independently of whether trial publications used all of the data or only a fraction. We will prefer datasets without imputed missing data.

Participant-level data characteristics will be checked as follows using structured forms. IPD from each trial will be checked for missing participants (e.g. compare IPD samples against trial CONSORT diagrams to ensure that IPD from all randomised participants is included if available), for missing outcomes and missing pre-specified effect modifiers (compared against trial publications and protocols), and for invalid, out of range or inconsistent items (e.g. unusually old or young participants), before being converted to standard format. We will request individual items from questionnaires, recalculating scale-specific scores where possible. We will check with trial authors whether any questionnaire items had been reversed, if applicable. IPD will be cross-examined against the summary statistics reported in trial publications. Inconsistencies will be checked by another reviewer. If they confirm that the numbers do not match, we will attempt to explain the difference (e.g. the publication may have used a per-protocol sample and we may have used the full randomized sample), and we will contact trial authors for clarification until inconsistencies are understood, and corrected if applicable.

Risk of bias assessment

Two reviewers will independently assess newly-found trials' risk of bias using the revised Cochrane risk-of-bias tool (RoB2) for randomised trials applied to the outcomes included in this review [46 47]. This tool stringently measures potential bias across five sources: (1) randomisation, (2) deviations from intended interventions, (3) missing outcome data, (4) measurement of the outcome, and (5) selection of the reported result. We will resolve discrepancies through discussion, involving a third reviewer where necessary.

Our previous review has found that many trials have high risk of bias from several sources, reducing confidence in the cumulative evidence. To understand how results were affected, we performed a sensitivity analysis removing trials deemed to be at high risk of bias from three or more sources, which divided the sample into roughly equal parts. The sensitivity analysis showed that the results were generally sensitive to this criterion. Accordingly, to maximize confidence in the IPD meta-analysis results and to maintain consistency with our previous review, we plan to only include trials

For peer review only - http://bmjopen.bmj.com/site/about/guidelines.xhtml

with a maximum of two high risk-of-bias domains, as assessed before obtaining IPD. These are the trials likely to provide the most reliable evidence in the field.

We acknowledge, however, that this criterion is sub-optimal, potentially imposing a limitation on our findings. The RoB2 tool has not been validated as a scale, so there are no validated cut-off points and domains may not be interchangeable [48]. Therefore, the included studies may have very different types of flaws. These flaws will be described through a detailed assessment of the risks of bias of each of the included studies using the RoB2 tool. We will also discuss our findings in relation to the sensitivity analysis performed in our previous review.

Once studies have been selected and IPD obtained, risk of bias for individual studies will be updated according to the IPD available (e.g. risk lowered if IPD includes participants missing in published trial reports). We will check allocation for any unusual patterns. When key aspects are unclear, we will seek information from study authors. In order to assess the confidence in the cumulative evidence, we will use the GRADE approach (Guyatt et al. 2008).

Effect measures

The main outcome will be self-reported psychological distress measured between one and six months after programme completion using psychometrically valid questionnaires scored on a continuous scale (e.g. Perceived Stress Scale, General Health Questionnaire, Depression, Anxiety and Stress Scale). Questionnaires asking about fleeting states (e.g. "How do you feel now?") will be excluded.

Post-intervention psychological distress measures (i.e. those taken less than one month after programme completion) will be grouped and considered as a secondary outcome: they do not inform stable changes, therefore are less useful for understanding the real-life impact of MBPs. Psychological distress follow-up measured beyond 6 months will be grouped and also considered as a secondary outcome. If a study measured the outcome more than once within the time point range of interest, the longest follow-up will be used.

We expect that trials will use different questionnaires to measure psychological distress, therefore we will standardise them using z-scores. . We will calculate the the ANCOVA estimate (final score adjusted for baseline score) and adjust it for the available pre-specified effect modifiers [49 50].

If a trial reports more than one psychological distress measure within the same time point range, we will prefer the one assigned as primary outcome by the trialists; if this is not stated or none are primary outcomes, the one with best psychometric properties; if they have similar properties, the one that is used most frequently in the other studies. Full questionnaire scales and untransformed data will be preferred.

Data synthesis

Although this project focuses on the effect moderator analyses, overall effects will be calculated and will be compared with those found in our previous review. Data synthesis will be quantitative.. Two-stage IPD meta-analyses will be performed, as they automatically stratify parameter estimates by trial, use well-known meta-analysis methods, are more transparent than one-stage methods, and easily enable forest plots [49]. . They will be univariate for the time-point ranges for which data from all the trials are available, otherwise they will be multivariate and include all available time-point ranges [51].

For peer review only - http://bmjopen.bmj.com/site/about/guidelines.xhtml

Stage one of the two-stage IPD meta-analyses will involve conducting linear regressions separately by trial to estimate the trial's intervention effects. The models will include the baseline measurement of the outcome and the pre-specified effect modifiers available for that trial [49]. Stage two will combine the intervention effects from each trial using pairwise random-effects meta-analyses (a common effect is highly implausible) within comparator categories.

The main analysis will compare MBPs with a combination of all the passive control groups. If the included trials also compared MBPs with other interventions, these will be grouped under the comparator 'active control', and effects will be explored for this comparison in secondary analyses. In the event of finding multi-armed trials with multiple control groups that fit one category, these control groups will be combined. Two-arm trials that compare two eligible MBPs with each other will not be included. In multi-arm trials that do this, the two MBP arms will be combined for meta-analysis.

Estimation of heterogeneity will be performed using restricted maximum likelihood. To quantify the heterogeneity in the intervention effect, approximate prediction intervals will be calculated [52]. Intention-to-treat analyses of individual trials will be conducted for verification, to compare against published analyses and to discuss reasons for potential differences. Trials for which IPD are not made available will be included in a sensitivity analysis incorporating the available aggregate data. Results will be compared with IPD-data-only results [16].

Multiple imputation strategies will be applied to missing data (details in Appendix 3 of the online supplementary materials) [53 49 54]. A sensitivity analysis will compare results of imputed datasets with observed datasets. We will assess departures of the data missing at random assumption in sensitivity analyses at the individual study level, modelling missing data as 10% and 20% worse psychological stress scores than observed data. We will also explore the scenarios of missing distress scores in the intervention arm being worse than passive control group scores. In the mindfulness group participants who felt worse may have been less willing to answer because they were expecting an improvement or thought that they had done something wrong. Instead, passive control group participants may have expected to feel worse. We will explore how much worse missing outcome scores in the mindfulness arms would need to be for the significance and direction of the intervention effect to change.

Moderator analyses

The main moderator analyses will look at the effect of the moderators of interest one by one; if multiple interaction effects are found we will explore multivariable options to adjust for confounding as a secondary analysis. For each of the main moderator analyses, a treatment by participant covariate interaction term will be incorporated in the intervention effect trial regression models (first stage of two-stage meta-analysis), and the estimated interactions will be combined in a random effects meta-analysis. This method, known as the "deft" approach, will account for clustering of participants and separate out within-study and across-study information, avoiding ecological bias [55 56].

We will estimate subgroup-specific intervention effects by repeating the analysis procedure with the interaction parameters fixed at their "deft" estimates. Trials and/or individuals with missing values on an effect modifier will be excluded from the estimation of that interaction. If we find interaction effects after confounding adjustment, we will present a predictive model. We will test whether there is evidence of non-linear effects; if we find such evidence we will explore nonlinear models [55 57].

For peer review only - http://bmjopen.bmj.com/site/about/guidelines.xhtml

Continuous variables will not be categorised for analysis. We expect that trials will use different questionnaires to measure baseline psychological distress and dispositional mindfulness; we will standardise them using z-scores. Education level data are usually collected in the form of categories with a natural ordering; if that is the case then a linear trend across categories will be assumed [56]. Where trials have used different categories for collecting education level data, we shall strive to retain an ordered-categorical approach where levels have been collapsed by, for instance, PhD=1, BA=2, PhD/BA=1.5, or by estimating years of education. Genders other than man/woman will be combined into an "other" category.

ETHICS AND DISSEMINATION

No local ethics approval was deemed necessary for this project following consultation with the research governance team. Trial authors will be requested to anonymise data sets prior to sharing them, and asked to confirm they have obtained ethical approval for sharing trial data anonymously. Data management and analysis will take place at the Department of Psychiatry, University of Cambridge. Data as obtained from individual trial authors will be stored at the highly secure Clinical School Secure Data Hosting Service and checked for any residual identifiable data before making copies to be used in normal workstations. The aggregate data and analysis code will be shared in a public repository.

Findings will be disseminated within the academic community through publication in scientific journals, conference presentations and networking. Professional stakeholders will be reached through activities focused on discussing the applicability of the findings. Media channels, social media (@MSSatUoC), and a variety of presentation formats will be used to engage with the wider public.

PATIENT AND PUBLIC INVOLVEMENT

A public stakeholder group is providing input throughout the life of this project. Members bring experiential expertise on mindfulness' effects and how they interact with contextual or personal factors, and on mental health promotion in daily life. We train and support them so that they are able to conceptually understand the study and can co-produce it. Stakeholders shaped the research questions and prioritised outcomes and moderation analyses. They will be invited to contribute to the day-to-day research process as research partners, for example by selecting studies and extracting data. They will be involved in interpreting the results, creating an impact plan, and disseminating the findings. We are also involving a group of professional stakeholders.

ACKNOWLEDGEMENTS

We are extremely grateful to those in our professional and public stakeholder groups for their keen involvement.

For peer review only - http://bmjopen.bmj.com/site/about/guidelines.xhtml

AUTHORS' CONTRIBUTIONS

JG applied for research funding and is the guarantor. JG, PBJ, TD and IRW planned the study. JG and CF wrote the manuscript that was revised through discussion with all the authors. All authors read and approved the final manuscript.

FUNDING STATEMENT

This publication presents independent research funded by the National Institute for Health Research (NIHR). The views expressed are those of the authors and not necessarily those of the NHS, the NIHR or the Department of Health and Social Care. JG is funded by a NIHR Post-doctoral Fellowship for this research project (salary and all project costs, PDF-2017-10-018, https://www.nihr.ac.uk/). CF's salary for this research project was funded by a Cambridgeshire & Peterborough NHS Foundation Trust grant awarded to JG (RNAG/552, https://www.cpft.nhs.uk/). IRW was supported by the UK Medical Research Council (MC_UU_12023/21, https://mrc.ukri.org/). TD was supported by the UK Medical Research Council (SUAG/043 G101400, https://mrc.ukri.org/), the Wellcome Trust (104908/Z/14/Z, 107496/Z/15/Z, https://wellcome.org/), and the NIHR Cambridge Biomedical Research Centre (RG85446, 247730, https://cambridgebrc.nihr.ac.uk/). PBJ is supported by the Wellcome Trust (095844/Z/11/Z, https://wellcome.org/), the UK Medical Research Council (MR/N019067/1, https://mrc.ukri.org/), and the NIHR ARC East of England (RNAG/564, https://arc-eoe.nihr.ac.uk/). The funders had no role in study design, data collection and analysis, decision to publish, or preparation of this protocol.

COMPETING INTERESTS STATEMENT

The authors have no conflicts of interest to declare.

For peer review only

For peer review only - http://bmjopen.bmj.com/site/about/guidelines.xhtml

REFERENCES

1. Vos T, Barber RM, Bell B, et al. Global, regional, and national incidence, prevalence, and years lived with disability for 301 acute and chronic diseases and injuries in 188 countries, 1990-2013: a systematic analysis for the Global Burden of Disease Study 2013. *The Lancet* 2015;386(9995):743-800. doi: 10.1016/S0140-6736(15)60692-4
2. Vindegaard N, Benros ME. COVID-19 pandemic and mental health consequences: Systematic review of the current evidence. *Brain Behav Immun* 2020;89:531-42. doi: 10.1016/j.bbi.2020.05.048 [published Online First: 2020/06/03]
3. Samele C. Increasing momentum in prevention of mental illness and mental health promotion across Europe. *BJPsych International* 2016;13(1):22-23.
4. Russ TC, Stamatakis E, Hamer M, et al. Association between psychological distress and mortality: individual participant pooled analysis of 10 prospective cohort studies. *BMJ (Clinical research ed)* 2012;345:e4933. doi: 10.1136/bmj.e4933 [published Online First: 2012/08/02]
5. Kabat-Zinn J. Foreword: Seeds of a necessary global renaissance in the making: the refining of psychology's understanding of the nature of mind, self, and embodiment through the lens of mindfulness and its origins at a key inflection point for the species. *Current opinion in psychology* 2019;28:xi-xvii. doi: 10.1016/j.copsyc.2019.02.005 [published Online First: 2019/05/18]
6. Burke A, Lam CN, Stussman B, et al. Prevalence and patterns of use of mantra, mindfulness and spiritual meditation among adults in the United States. *BMC complementary and alternative medicine* 2017;17(1):316. doi: 10.1186/s12906-017-1827-8 [published Online First: 2017/06/18]
7. Barnes N, Hattan P, Black DS, et al. An Examination of Mindfulness-Based Programs in US Medical Schools. *Mindfulness* 2017;8(2): 489–94. doi: 10.1007/s12671-016-0623-8
8. Fortune. Meditation Has Become A Billion-Dollar Business 2016 [Available from: http://fortune.com/2016/03/12/meditation-mindfulness-apps/ accessed 01/12/2016.
9. Kabat-Zinn J. Full Catastrophe Living, Revised Edition: How to cope with stress, pain and illness using mindfulness meditation. 2 ed. London: Piatkus 2013.
10. Crane RS, Brewer J, Feldman C, et al. What defines mindfulness-based programs? The warp and the weft. *Psychol Med* 2017;47(6):990-99. doi: 10.1017/S0033291716003317 [published Online First: 2016/12/30]
11. Galante J, Friedrich C, Dawson AF, et al. Mindfulness-based programmes for mental health promotion in adults in non-clinical settings: A systematic review and meta-analysis of randomised controlled trials. *PLOS Medicine* 2021;18(1):e1003481. doi: https://doi.org/10.1371/journal.pmed.1003481
12. Goldberg SB, Sun S, Davidson RJ. The empirical status of mindfulness based interventions: A systematic review of 44 meta-analyses of randomized controlled trials. *Perspectives on Psychological Science* 2020
13. De Vibe M. Mindfulness Based Stress Reduction (MBSR) for Improving Health, Quality of Life, and Social Functioning in Adults. *Campbell Systematic Reviews, DOI 104073/csr20123* 2012 doi: 10.4073/csr.2012.3
14. Hingorani AD, Windt DA, Riley RD, et al. Prognosis research strategy (PROGRESS) 4: stratified medicine research. *BMJ (Clinical research ed)* 2013;346:e5793. doi: 10.1136/bmj.e5793 [published Online First: 2013/02/07]
15. Ioannidis JPA, Lau J. Uncontrolled Pearls, Controlled Evidence, Meta-Analysis and the Individual Patient. *Journal of clinical epidemiology* 1998;51(8):709-11.
16. Riley RD, Lambert PC, Abo-Zaid G. Meta-analysis of individual participant data: rationale, conduct, and reporting. *BMJ (Clinical research ed)* 2010;340:c221. doi: 10.1136/bmj.c221 [published Online First: 2010/02/09]

For peer review only - http://bmjopen.bmj.com/site/about/guidelines.xhtml

17. Keng SL, Smoski MJ, Robins CJ. Effects of mindfulness on psychological health: a review of empirical studies. *Clinical psychology review* 2011;31(6):1041-56. doi: 10.1016/j.cpr.2011.04.006 [published Online First: 2011/08/02]
18. Davidson RJ. Mindfulness-Based Cognitive Therapy and the Prevention of Depressive Relapse: Measures, Mechanisms, and Mediators. *JAMA psychiatry* 2016;73(6):547-8. doi: 10.1001/jamapsychiatry.2016.0135 [published Online First: 2016/04/28]
19. Ospina MB, Bond K, Karkhaneh M, et al. Meditation practices for health: state of the research. *Evidence report/technology assessment* 2007(155):1-263. [published Online First: 2007/09/04]
20. Rojiani R, Santoyo JF, Rahrig H, et al. Women Benefit More Than Men in Response to College-based Meditation Training. *Frontiers in psychology* 2017;8 doi: 10.3389/fpsyg.2017.00551
21. Shapiro SL, Brown KW, Thoresen C, et al. The moderation of Mindfulness-based stress reduction effects by trait mindfulness: results from a randomized controlled trial. *Journal of clinical psychology* 2011;67(3):267-77. doi: 10.1002/jclp.20761 [published Online First: 2011/01/22]
22. Schellekens MPJ, van den Hurk DGM, Prins JB, et al. Mindfulness-based stress reduction added to care as usual for lung cancer patients and/or their partners: A multicentre randomized controlled trial. *Psycho-oncology* 2017;26(12):2118-26. doi: 10.1002/pon.4430
23. Hofmann SG, Sawyer AT, Witt AA, et al. The effect of mindfulness-based therapy on anxiety and depression: A meta-analytic review. *J Consult Clin Psychol* 2010;78(2):169-83. doi: 10.1037/a0018555 [published Online First: 2010/03/31]
24. Roos CR, Bowen S, Witkiewitz K. Baseline patterns of substance use disorder severity and depression and anxiety symptoms moderate the efficacy of mindfulness-based relapse prevention. *Journal of consulting and clinical psychology* 2017;85(11):1041-51. doi: 10.1037/ccp0000249
25. Khoury B, Lecomte T, Fortin G, et al. Mindfulness-based therapy: a comprehensive meta-analysis. *Clinical psychology review* 2013;33(6):763-71. doi: 10.1016/j.cpr.2013.05.005 [published Online First: 2013/06/26]
26. Galante J, Stochl J, Dufour G, et al. Effectiveness of providing university students with a mindfulness-based intervention to increase resilience to stress: one-year follow-up of a pragmatic randomised controlled trial. *Journal of Epidemiology and Community Health* 2020;75:151-60. doi: http://dx.doi.org/10.1136/jech-2020-214390
27. Kuyken W, Warren FC, Taylor RS, et al. Efficacy of Mindfulness-Based Cognitive Therapy in Prevention of Depressive Relapse: An Individual Patient Data Meta-analysis From Randomized Trials. *JAMA psychiatry* 2016;73(6):565-74. doi: 10.1001/jamapsychiatry.2016.0076 [published Online First: 2016/04/28]
28. Fiocco AJ, Mallya S, Farzaneh M, et al. Exploring the benefits of mindfulness training in healthy community-dwelling older adults: A randomized controlled study using a mixed methods approach. *Mindfulness* 2018;10(4):737-48. doi: 10.1007/s12671-018-1041-x
29. Vonderlin R, Biermann M, Bohus M, et al. Mindfulness-Based Programs in the Workplace: a Meta-Analysis of Randomized Controlled Trials. *Mindfulness* 2020;11:1579–98. doi: 10.1007/s12671-020-01328-3
30. Galante J, Dufour G, Vainre M, et al. A mindfulness-based intervention to increase resilience to stress in university students (the Mindful Student Study): a pragmatic randomised controlled trial. *The Lancet Public Health* 2018;3(2):e72-e81. doi: 10.1016/s2468-2667(17)30231-1
31. De Vibe M, Solhaug I, Tyssen R, et al. Mindfulness training for stress management: a randomised controlled study of medical and psychology students. *BMC Medical Education* 2013;13(1):107.
32. Dunning DL, Griffiths K, Kuyken W, et al. The Effects of Mindfulness-Based Interventions on Cognition and Mental Health in Children and Adolescents: A Meta-Analysis of Randomised Controlled Trials. *J Child Psychol Psychiatry* 2019;60(3):244–58.

For peer review only - http://bmjopen.bmj.com/site/about/guidelines.xhtml

33. Dawson AF, Brown WW, Anderson J, et al. Mindfulness-based Interventions for University Students: A Systematic Review and Meta-analysis of Randomized Controlled Trials. *Applied Psychology: Health and Well-Being* 2020;12(2):384-410.
34. Nyklicek I, Irrmischer M. For Whom Does Mindfulness-Based Stress Reduction Work? Moderating Effects of Personality. *Mindfulness (N Y)* 2017;8(4):1106-16. doi: 10.1007/s12671-017-0687-0 [published Online First: 2017/08/02]
35. Greeson JM, Smoski MJ, Suarez EC, et al. Decreased Symptoms of Depression After Mindfulness-Based Stress Reduction: Potential Moderating Effects of Religiosity, Spirituality, Trait Mindfulness, Sex, and Age. *The Journal of Alternative and Complementary Medicine* 2015;21(3):166-74. doi: 10.1089/acm.2014.0285
36. Ebert DD, Donkin L, Andersson G, et al. Does Internet-based guided-self-help for depression cause harm? An individual participant data meta-analysis on deterioration rates and its moderators in randomized controlled trials. *Psychological Medicine* 2016;46(13):2679-93. doi: 10.1017/s0033291716001562
37. Russell L, Ugalde A, White V, et al. Relevance of mindfulness practices for culturally and linguistically diverse cancer populations. *Psycho-oncology* 2019;28(11):2250-52. doi: 10.1002/pon.5221 [published Online First: 2019/09/06]
38. Rau HK, Williams PG. Dispositional mindfulness: A critical review of construct validation research. *Personality and Individual Differences* 2016;93:32-43. doi: 10.1016/j.paid.2015.09.035
39. Gawrysiak MJ, Grassetti SN, Greeson JM, et al. The many facets of mindfulness and the prediction of change following mindfulness-based stress reduction (MBSR). *Journal of clinical psychology* 2018;74(4):523-35. doi: 10.1002/jclp.22521 [published Online First: 2017/08/18]
40. Shapiro SL, Brown KW, Thoresen C, et al. The moderation of Mindfulness-based stress reduction effects by trait mindfulness: Results from a randomized controlled trial. *Journal of clinical psychology* 2011;67(3):267-77. doi: https://doi.org/10.1002/jclp.20761
41. Burton H, Sagoo GS, Pharoah PDP, et al. Time to revisit Geoffrey Rose: strategies for prevention in the genomic era? *Italian Journal of Public Health* 2012;9(4):e8665-1. doi: 10.2427/8665
42. Tierney JF, Fisher DJ, Burdett S, et al. Comparison of aggregate and individual participant data approaches to meta-analysis of randomised trials: An observational study. *PLoS Med* 2020;17(1):e1003019. doi: 10.1371/journal.pmed.1003019 [published Online First: 2020/02/01]
43. Shamseer L, Moher D, Clarke M, et al. Preferred reporting items for systematic review and meta-analysis protocols (PRISMA-P) 2015: elaboration and explanation. *BMJ (Clinical research ed)* 2015;350:g7647. doi: 10.1136/bmj.g7647 [published Online First: 2015/01/04]
44. Mayo-Wilson E, Li T, Fusco N, et al. Cherry-picking by trialists and meta-analysts can drive conclusions about intervention efficacy. *Journal of clinical epidemiology* 2017;91:95-110. doi: 10.1016/j.jclinepi.2017.07.014 [published Online First: 2017/08/27]
45. Jayawardene WP, Lohrmann DK, Erbe RG, et al. Effects of preventive online mindfulness interventions on stress and mindfulness: A meta-analysis of randomized controlled trials. *Preventive Medicine Reports* 2017;5:150-59. doi: 10.1016/j.pmedr.2016.11.013
46. RoB2 Development Group. Revised Cochrane risk-of-bias tool for randomized trials (RoB 2) 2018 [updated 11 September 2018. Available from: https://sites.google.com/site/riskofbiastool/welcome/rob-2-0-tool/current-version-of-rob-2?authuser=0 accessed 21 September 2018
47. Sterne JAC, Savović J, Page MJ, et al. RoB 2: a revised tool for assessing risk of bias in randomised trials. *BMJ (Clinical research ed)* 2019;366:l4898. doi: 10.1136/bmj.l4898
48. Savovic J, Turner RM, Mawdsley D, et al. Association Between Risk-of-Bias Assessments and Results of Randomized Trials in Cochrane Reviews: The ROBES Meta-Epidemiologic Study. *American journal of epidemiology* 2018;187(5):1113-22. doi: 10.1093/aje/kwx344
49. Riley RD, Tierney JF, Stewart LA, editors. *Individual Participant Data Meta-Analysis: A Handbook for Healthcare Research*: Wiley, 2021.

For peer review only - http://bmjopen.bmj.com/site/about/guidelines.xhtml

50. McKenzie JE, Herbison GP, Deeks JJ. Impact of analysing continuous outcomes using final values, change scores and analysis of covariance on the performance of meta-analytic methods: a simulation study. *Research synthesis methods* 2016;7(4):371-86. doi: 10.1002/jrsm.1196 [published Online First: 2015/12/31]
51. Mavridis D, Salanti G. A practical introduction to multivariate meta-analysis. *Stat Methods Med Res* 2013;22(2):133-58. doi: 10.1177/0962280211432219 [published Online First: 2012/01/26]
52. Riley RD, Higgins JPT, Deeks JJ. Interpretation of random effects meta-analyses. *BMJ (Clinical research ed)* 2011;342:d549.
53. Debray TP, Moons KG, van Valkenhoef G, et al. Get real in individual participant data (IPD) meta-analysis: a review of the methodology. *Research synthesis methods* 2015;6(4):293-309. doi: 10.1002/jrsm.1160 [published Online First: 2015/08/20]
54. White IR, Royston P, Wood AM. Multiple imputation using chained equations: Issues and guidance for practice. *Statistics in medicine* 2011;30(4):377-99. doi: 10.1002/sim.4067 [published Online First: 2011/01/13]
55. Riley RD, Debray TPA, Fisher D, et al. Individual participant data meta-analysis to examine interactions between treatment effect and participant-level covariates: Statistical recommendations for conduct and planning. *Statistics in medicine* 2020;39(15):2115-37. doi: 10.1002/sim.8516 [published Online First: 2020/05/01]
56. Fisher DJ, Carpenter JR, Morris TP, et al. Meta-analytical methods to identify who benefits most from treatments: daft, deluded, or deft approach? *BMJ (Clinical research ed)* 2017;356:j573. doi: 10.1136/bmj.j573 [published Online First: 2017/03/05]
57. White IR, Kaptoge S, Royston P, et al. Meta-analysis of non-linear exposure-outcome relationships using individual participant data: A comparison of two methods. *Statistics in medicine* 2019;38(3):326-38. doi: 10.1002/sim.7974 [published Online First: 2018/10/05]
58. Gu J, Strauss C, Bond R, et al. How do mindfulness-based cognitive therapy and mindfulness-based stress reduction improve mental health and wellbeing? A systematic review and meta-analysis of mediation studies. *Clinical psychology review* 2015;37C:1-12. doi: 10.1016/j.cpr.2015.01.006 [published Online First: 2015/02/18]
59. Klatt MD, Buckworth J, Malarkey WB. Effects of low-dose mindfulness-based stress reduction (MBSR-ld) on working adults. *Health Education and Behavior* 2009;36(3):601-14. doi: http://dx.doi.org/10.1177/1090198108317627

For peer review only - http://bmjopen.bmj.com/site/about/guidelines.xhtml

TABLES

TABLE 1. REVIEW INCLUSION CRITERIA

Study aspect	Inclusion criterion
Design	Parallel-arm randomised controlled trials (RCTs) including cluster RCTs
Intervention	Group-based first generation MBPs as defined by Crane et al [10], with a minimum intensity of four one-hour in-person teacher-led sessions or equivalent*.
Comparison	Passive control groups such as no intervention, waitlists, or treatment-as-usual if the MBP arm also had access to it.
Population	Adult (18+ year old) participants living in the community, as long as the trial had not selected them for having any particular clinical condition. MBPs targeting specific community groups were included. Trials with slightly younger participants (e.g. those in university settings where some students will turn 18 during the first academic year) will be included.
Outcomes	Self-reported psychological distress measured between one and six months after MBP completion.
Effect modifiers	At least one of the following has been measured: baseline psychological distress, gender, age, education, and dispositional mindfulness.
Quality	A maximum of two high risk-of-bias sources as assessed using the RoB2 tool [47] before obtaining IPD (rationale in "Risk of Bias Assessment" section)

* Four MBP sessions were used as the "minimum dose" for participants in previous studies [58], and one-hour sessions are common in non-clinical settings [59]

For peer review only

Mindfulness-based programmes for mental health promotion in adults in non-clinical settings: protocol of an individual participant data meta-analysis of randomised controlled trials

Online Supplementary Material

Appendix 1 - Search strategy for EMBASE (Ovid interface)

S1 exp meditation/ or exp mindfulness/
S2 (mindfullness or mindfulness or meditat*).ab. or (mindfullness or mindfulness or meditat*).ti.
S3 1 or 2
S4 clinical trial/
S5 randomized controlled trial/
S6 controlled clinical trial/
S7 multicenter study/
S8 phase 3 clinical trial/
S9 phase 4 clinical trial/
S10 double blind procedure/
S11 placebo/ S12 exp randomization/
S13 (randomi?ed controlled trial$ or rct or (random$ adj2 allocat$) or single blind$ or double blind$ or ((treble or triple) adj blind$) or placebo$).tw.
S14 Prospective Study.mp. or prospective study/
S15 4 or 5 or 6 or 7 or 8 or 9 or 10 or 11 or 12 or 13 or 14
S16 3 and 15

For peer review only - http://bmjopen.bmj.com/site/about/guidelines.xhtml

Appendix 2 – Aggregate data extraction form

Categories	Extracted information
Study Identification	Sponsorship sources, conflicts of interest, country, study setting, corresponding authors, institution, emails, date recruitment started, and year first published.
Methods	Study design, conceptualisation of mindfulness, incentives for participants, number of participants (total randomised and per arm), and power calculation.
Population	Inclusion and exclusion criteria, type of participant, group differences, baseline characteristics
Interventions	Intervention name, mention of intervention manual, whether it was an adaptation of another intervention (rationale), intensity, mindfulness components (type, frequency and duration), non-mindfulness components (type, frequency and duration), home practice length and type, group size, any individual tailoring, any booster sessions or support after the end of the programme, adherence to intervention manual, intervention setting, teacher competence, teacher characteristics, response to intervention (attendance, satisfaction, reasons for missing sessions), and whether participants paid to do the course.
Outcomes	Outcome measure used, time points, group sizes, effect measures available and extracted effect sizes.

For peer review only

Appendix 3 – Multiple imputation plan details

All imputations will be done separately by trial. This is compatible with a two-stage meta-analysis where results from imputed data sets are combined by Rubin's rules to give a trial-specific estimate ready for meta-analysis.

All imputations will also be done separately by randomised group. This respects all possible treatment-by-covariate interactions. If the trial is a cluster-RCT, clustering will be ignored in the imputation but allowed for in the analysis.

For each trial, individuals with all missing outcomes will be dropped before imputation, and the remaining missing outcomes will be multiply imputed either item by item or as a total score. If for *most* (>50%) individuals most (>50%) of the questionnaire items are available, we will impute the items of that questionnaire *for all of the individuals* in the trial; if not, we will impute total scores. We will use predictive mean matching.

All outcomes will be included and imputed in the same model (multivariate imputation by chained equations, MICE) because outcomes will act as auxiliary variables for each other. All of the moderators will be included and imputed in the same model so that they contribute to imputing the outcomes, but observations with imputed moderators will be dropped for analysis. We will choose a number of imputations at least equal to the percentage of incomplete cases.

For peer review only

For peer review only - http://bmjopen.bmj.com/site/about/guidelines.xhtml

PRISMA-P (Preferred Reporting Items for Systematic review and Meta-Analysis Protocols) 2015 checklist: recommended items to address in a systematic review protocol*

Section and topic	Item No	Checklist item	Reported
**ADMINISTRATIVE INFORMATION**			
**Title:**			
Identification	1a	Identify the report as a protocol of a systematic review	*p.1*
Update	1b	If the protocol is for an update of a previous systematic review, identify as such	*Not applicable*
**Registration**	2	If registered, provide the name of the registry (such as PROSPERO) and registration number	*p. 2 and 6*
**Authors:**			
Contact	3a	Provide name, institutional affiliation, e-mail address of all protocol authors; provide physical mailing address of corresponding author	*p. 1*
Contributions	3b	Describe contributions of protocol authors and identify the guarantor of the review	*p. 10*
**Amendments**	4	If the protocol represents an amendment of a previously completed or published protocol, identify as such and list changes; otherwise, state plan for documenting important protocol amendments	*Not applicable*
**Support:**			
Sources	5a	Indicate sources of financial or other support for the review	*p. 10 and 11*
Sponsor	5b	Provide name for the review funder and/or sponsor	*p. 10*
Role of sponsor or funder	5c	Describe roles of funder(s), sponsor(s), and/or institution(s), if any, in developing the protocol	*p. 10*
**INTRODUCTION**			
**Rationale**	6	Describe the rationale for the review in the context of what is already known	*p. 4*
**Objectives**	7	Provide an explicit statement of the question(s) the review will address with reference to participants, interventions, comparators, and outcomes (PICO)	*p. 6*
**METHODS**			
**Eligibility criteria**	8	Specify the study characteristics (such as PICO, study design, setting, time frame) and report characteristics (such as years considered, language, publication status) to be used as criteria for eligibility for the review	*p. 16*
**Information sources**	9	Describe all intended information sources (such as electronic databases, contact with study authors, trial registers or other grey literature sources) with planned dates of coverage	*p. 6*

For peer review only

**Search strategy**	10	Present draft of search strategy to be used for at least one electronic database, including planned limits, such that it could be repeated	*Appendix p. 1*
**Study records:**			
Data management	11a	Describe the mechanism(s) that will be used to manage records and data throughout the review	*p. 6, 7 and 10*
Selection process	11b	State the process that will be used for selecting studies (such as two independent reviewers) through each phase of the review (that is, screening, eligibility and inclusion in meta-analysis)	*p. 6*
Data collection process	11c	Describe planned method of extracting data from reports (such as piloting forms, done independently, in duplicate), any processes for obtaining and confirming data from investigators	*p. 7*
Data items	12	List and define all variables for which data will be sought (such as PICO items, funding sources), any pre-planned data assumptions and simplifications	*p. 7, Appendix p. 1*
**Outcomes and prioritization**	13	List and define all outcomes for which data will be sought, including prioritization of main and additional outcomes, with rationale	*p. 8*
**Risk of bias in individual studies**	14	Describe anticipated methods for assessing risk of bias of individual studies, including whether this will be done at the outcome or study level, or both; state how this information will be used in data synthesis	*p. 7*
**Data synthesis**	15a	Describe criteria under which study data will be quantitatively synthesised	*p. 8 and 9*
	15b	If data are appropriate for quantitative synthesis, describe planned summary measures, methods of handling data and methods of combining data from studies, including any planned exploration of consistency (such as I^2, Kendall's τ)	*p. 8 and 9*
	15c	Describe any proposed additional analyses (such as sensitivity or subgroup analyses, meta-regression)	*p. 9*
	15d	If quantitative synthesis is not appropriate, describe the type of summary planned	*Not applicable*
**Meta-bias(es)**	16	Specify any planned assessment of meta-bias(es) (such as publication bias across studies, selective reporting within studies)	*Not applicable*
**Confidence in cumulative evidence**	17	Describe how the strength of the body of evidence will be assessed (such as GRADE)	*p. 8*

From: Shamseer L, Moher D, Clarke M, Ghersi D, Liberati A, Petticrew M, Shekelle P, Stewart L, PRISMA-P Group. Preferred reporting items for systematic review and meta-analysis protocols (PRISMA-P) 2015: elaboration and explanation. BMJ. 2015 Jan 2;349(jan02 1):g7647.

For peer review only
